# Design, synthesis and biological evaluation of novel tetrahydrothieno [2,3-c]pyridine substitued benzoyl thiourea derivatives as PAK1 inhibitors in triple negative breast cancer

**DOI:** 10.1080/14756366.2020.1797710

**Published:** 2020-08-04

**Authors:** Dahong Yao, Jian Huang, Jinhui Wang, Zhendan He, Jin Zhang

**Affiliations:** a School of Pharmaceutical Sciences, Shenzhen Technology University, Shenzhen, China; b School of Pharmaceutical Sciences, Guangdong Key Laboratory for Genome Stability and Human Disease Prevention, Shenzhen University Health Science Center, Shenzhen University, Shenzhen, China; c Drug Development Department, Shenzhen Honghui Bio-Pharmaceutical Co. Ltd., Shenzhen, China; d Collaborative Innovation Center for Biotherapy, West China Hospital, Sichuan University, Chengdu, China

**Keywords:** High-throughput virtual screening, PAK1 inhibitor, anti-proliferation, cell cycle arrests, breast cancer

## Abstract

The overexpression of P21-activated kinase 1 (PAK1) is associated with poor prognosis in several cancers, which has emerged as a promising drug targets. Based on high-throughput virtual screening strategy, tetrahydrothieno [2,3-c]pyridine scaffold was identified as an initial lead for targeting PAK1. Herein we reported our structure-based optimisation strategy to discover a potent PAK1 inhibitor (**7j**) which displayed potent PAK1 inhibition and antiproliferatory activity in MDA-MB-231 cells. **7j** induced obviously G2/M cell cycle arrest via PAK1-cdc25c-cdc2 pathway, and also inhibited MAPK-ERK and MAPK-JNK cascade to induce MDA-MB-231 cell death. Together, these results provided a novel chemical scaffold as PAK1 inhibitor for breast cancer treatment.

## 1. Introduction

P21-activated kinases (PAKs) belong to the STE20 family of serine/threonine kinases which is comprised of group I (PAK1, PAK2, and PAK3) and group II (PAK4, PAK5, and PAK6) based on sequence and structural homology^1^. PAKs functions as GTPase effector that links the Rho-related GTPases CDC42 and RAC1 to the JNK MAP kinase pathway that widely involve in cancer cell migration, proliferation, cell cycle and survival mechanisms^2–5^. Targeting p21-activated Kinase 1 Inhibits Growth and Metastasis via Raf1/MEK1/ERK Signalling in Oesophageal Squamous Cell Carcinoma Cells^6^. In particular, it was reported that *PAK1* gene amplification and protein overexpression were associated with poor prognosis in a variety of human cancers, including breast cancer^7^, Non-Small Cell Lung Cancer^8^, renal cell carcinoma^9^ and so forth. Furthermore, it was shown recently that combination of PAK1 inhibitor (FRAX1036) with taxane treatment could induce microtubule disorganisation, cell cycle arrests and cellular apoptosis in the luminal subtype of breast cancer^10^. PAK1 have emerged as a promising oncology targets and attracted a lot of pharmacologist interest due to its critical roles in cancers^11^.

**Figure 1.**
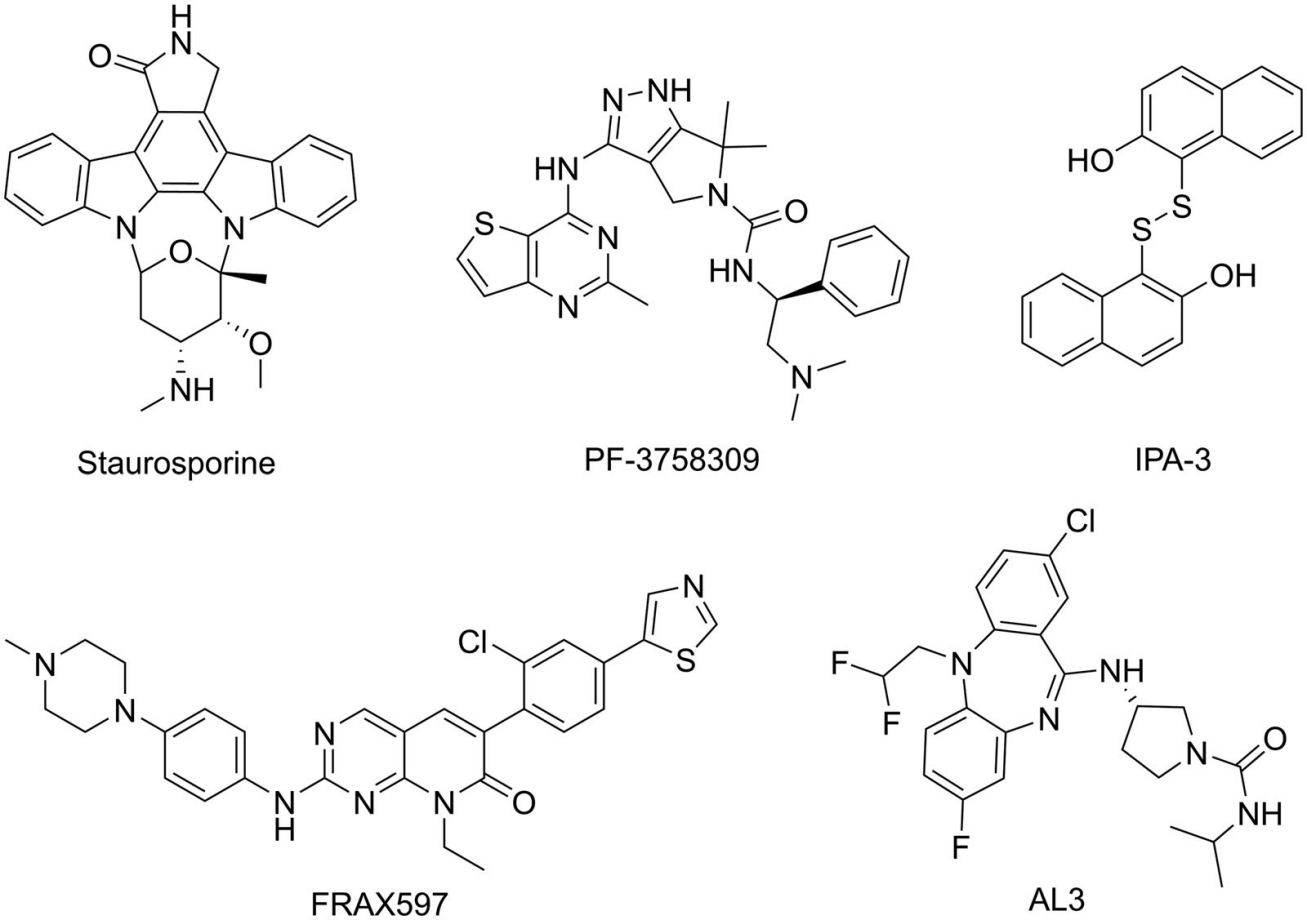
Some known PAK1 inhibitors.

Several PAK1 inhibitors have been described over the past few years (Figure 1). The ATP-competing PAK1 inhibitors have been extensively studied, but few chemical scaffolds, mainly including Oxindole/Maleimide-based inhibitors, such as **Staurosporine**^12^, Aminopyrazole-based inhibitors, such as **PF-3758309**^13^, and Aminopyrimidine-based inhibitors, such as **FRAX597**^14^. These ATP-competing inhibitors displayed high affinity and poor selectivity of PAK isoforms because of the similarity between the ATP-binding pockets of kinases. Recently, to achieve kinase selectivity, allosteric PAK1 inhibitors were designed and synthesised by targeting the specific site, such as **AL3**^15^ and **IPA-3**^16^. Unfortunately, to date only pan-PAK inhibitor **PF-35783099** progressed into clinical trials but is now stopped because of its poor potency *in vivo*. Consequently, there has been significant interest in the identification of potent PAK1 inhibitors with novel scaffold that are capability of clinical development for the breast cancers treatment.

In this study, we aimed to discover novel scaffolds with potential to optimise as potent PAK1 inhibitors. To achieve this, we identified the tetrahydrothieno[2,3-c]pyridine scaffold as a promising lead for targeting PAK1 by using a high-throughput virtual screening strategy. Subsequently, a series of tetrahydrothieno [2,3-c]pyridine substitued benzoyl thiourea derivatives was designed and synthesised based on structure-based strategy. A potent PAK1 inhibitor (**7j**) was discovered, which presented an IC_50_ value of 209 nM and inhibited MDA-MB-231 cell proliferation with an IC_50_ value of 4.67 μM. *In vitro*, **7j** could induce significant cell cycle arrest and cell death in MDA-MB-231 cells. Together, these results demonstrated that **7j** is a novel potent PAK1 inhibitor, which may provide a candidate drug for future cancer therapy.

## 2. Results and discussion

### 2.1. *The discovery of* tetrahydrothieno [2,3-c]pyridine *scaffold by virtual screening*

**Figure 2.**
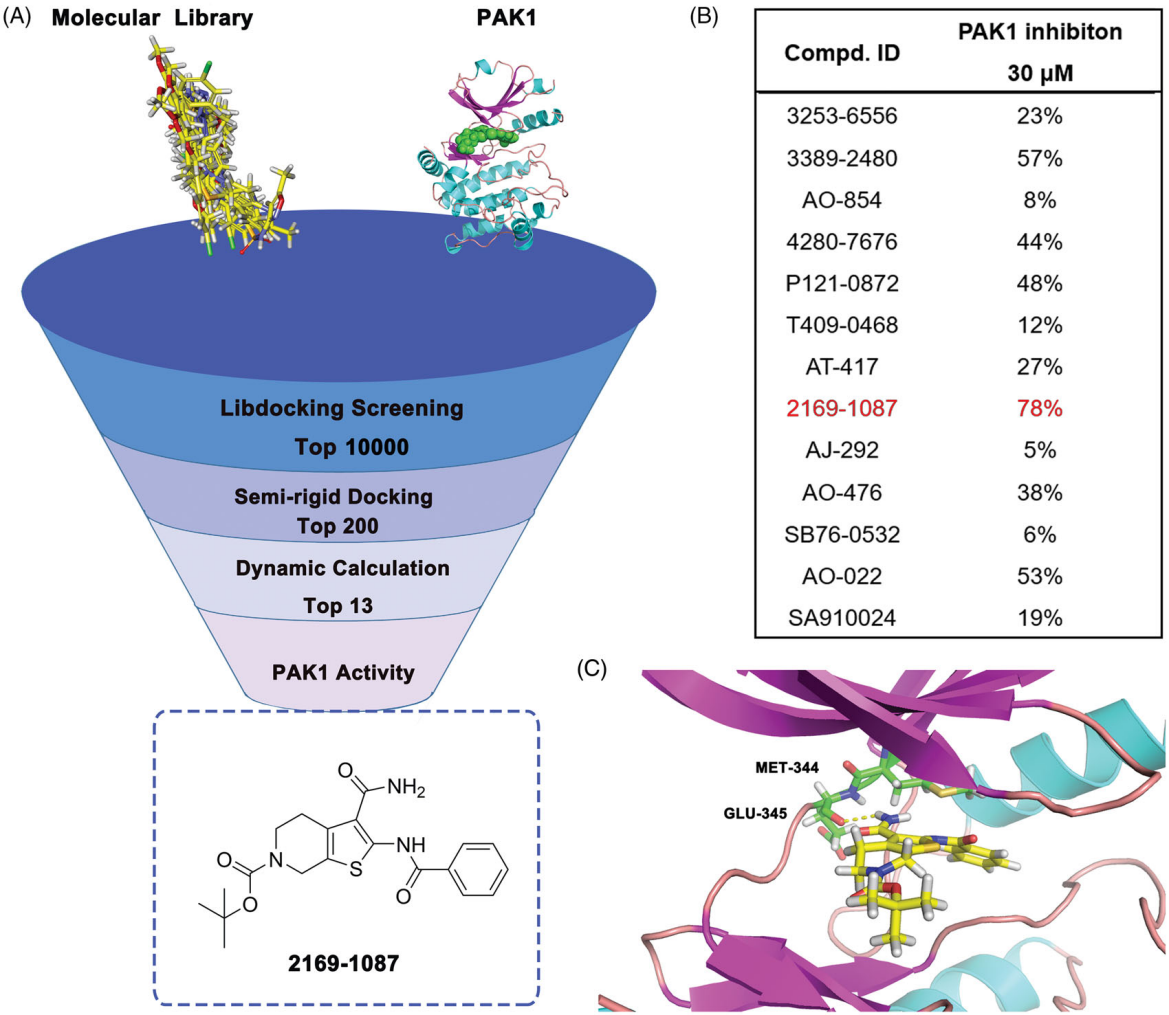
Virtual screening schematic model for the discovery process of novel PAK1 inhibitors.

In order to discover potential lead with novel skeleton, we adopt a comprehensive screening strategy combining virtual screening with enzymic analyses (Figure 2(A)). Firstly, we eliminated compounds with unfavourable druglike descriptors and physicochemical properties, the Chemdiv and Specs chemical library. Subsequently, these compounds were applied in structure-based pharmacophore (SBP) virtual screening through the Libdocking protocol in Discovery Studio 3.5 (DS). Only compounds ranked 10000 according to the fit scores were retained to access to next docking. The top 200 hits were obtained by a semi-rigid docking protocol. Additionally, molecular dynamics based on AMBER 10 were performed to further screen to product 13 hits carrying diverse scaffolds (Table S1). Subsequently, we conducted the PAK1 inhibitory activity assay to investigate the potency of these hits purchased commercially. The results showed that hit **2169–1087** with tetrahydrothieno[2,3-c]pyridine scaffold presented 78% inhibition rate against PAK1 at 30 μM (Figure 2(B)). Furthermore, we detected its half inhibitory concentration against PAK1, the result revealed that **2169–1087** showed a weak activity with IC_50_ value of 23.5 μM. In cell viability assay, **2169–1087** showed no antiproliferatory activity at 50 μM in MDA-MB-231 cells. In spite of this, we are not absolutely discouraged these poor results. In addition, we heavily analysed the binding poses of **2169–1087** and PAK1. As shown in Figure 2(C), 2169–1087 bound the ATP-binding site, and the formamide group at 3-site of thiophene was initiated a hydrogen bond with residue GLU345 located kinase hinge of ATP binding site. The benzoyl moiety at 2-site positioned the hydrophobic pocket II near the active loop of kinase, and the Boc-group located the entrance of kinase hinge by hydrophobic interaction. Collectively, **2169–1087** possessed the basic profiles serving as the frame to optimise PAK1 inhibitor. Structurally, a longer linker should be incorporated to make the hydrophobic group at 2-site of thiophene occupy the hydrophobic pocket II, and initiated hydrogen bond interactions with the gate control residues of the active loop. So benzoyl thiourea group was designed to serves as the linker and some hydrophobic groups were introduced to discuss the chemical space.

**Table 1.** The PAK1 inhibitory activity of compounds **5a–q**
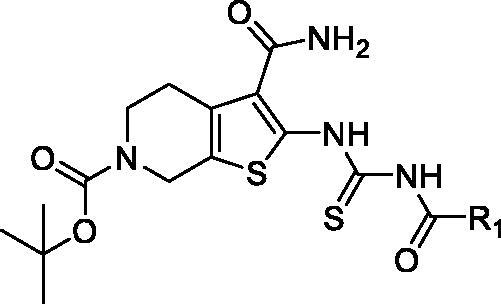

Compound	R_1_	Kinase inhibitory activity (IC_50_, μM)^a^
		PAK1
**5a**	phenyl	7.64 ± 0.72
**5b**	4-methylphenyl	7.28 ± 0.78
**5c**	2-methylphenyl	6.21 ± 0.69
**5d**	3-methylphenyl	4.32 ± 0.51
**5e**	2,4,6-trimethylphenyl	11.22 ± 1.01
**5f**	4-methoxyphenyl	16.38 ± 1.78
**5g**	4-bromophenyl	9.35 ± 0.97
**5h**	4-fluorophenyl	5.33 ± 0.65
**5i**	2-fluorophenyl	7.82 ± 0.71
**5j**	4-chlorophenyl	9.12 ± 0.95
**5k**	2-chlorophenyl	8.65 ± 0.93
**5l**	3-chlorophenyl	5.15 ± 0.54
**5m**	3-trifluoromethylphenyl	2.02 ± 0.27
**5n**	4-trifluoromethylphenyl	12.15 ± 1.35
**5o**	2,3-dichlorophenyl	7.43 ± 0.81
**5p**	cyclohexyl	19.33 ± 1.85
**5q**	2-thiophenemethyl	15.56 ± 1.64

^a^Each compound was tested in triplicate; the data are presented as the mean ± SD.

### 2.2. Chemistry

**Scheme 1.**
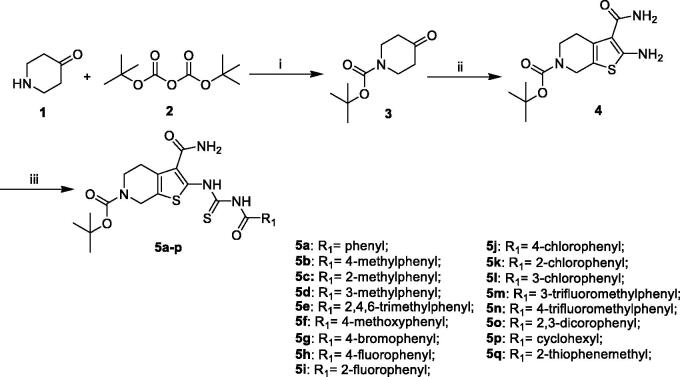
General synthesis of compounds **5a–q**. Reagents and conditions: (i) triethylamine, CH_2_Cl_2_, r.t., 16 h; (ii) S_8_, morpholine, EtOH, rt, 12 h; (iii) a. KSCN, Acyl chloride derivatives, acetonitrile, reflux, 6 h.

**Scheme 2.**
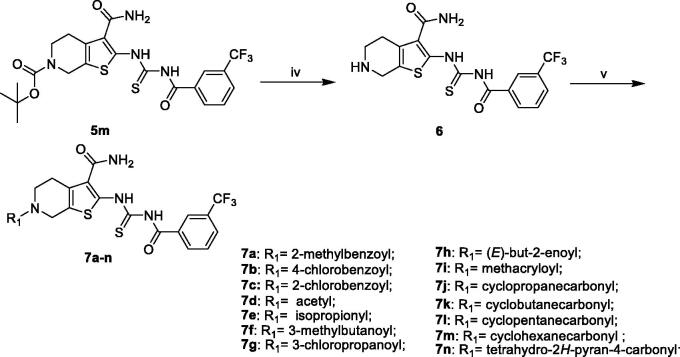
General synthesis of compounds **7a–n**. Reagents and conditions: (iv) TFA, CH_2_Cl_2_ r.t; (v) CH_2_Cl_2_, R_1_COCl, TEA, r.t.

The synthesis of compounds **5a–q** was carried out by using commercially available, piperidin-4-one (**1**) as the starting materials (Scheme 1). Piperidin-4-one was protected by *t*-butyloxycarboryl to yielded intermediate **3**. The Gewald reaction of intermediate **3** with S8 and morpholine gave intermediate **4**. Treatment of intermediate **4** with KSCN and Acyl chloride derivatives afforded compounds **5a–q**. Furthermore, the compounds **7a–n** were prepared from intermediate **5m** (Scheme 2). The synthesis of intermediate **6** was obtained by the deprotection of intermediate **5m** with TFA inCH_2_Cl_2_. Treatment of intermediate **6** with acyl chloride derivatives yielded compounds **7a–n**.

### 2.3. Analysis of the structure-activity relationship

To optimise the lead (**2169–1087**) based on the inhibitory activity against PAK1, we preferentially incorporated substituted-benzoyl thiourea to serve as the suitable linker, yielding compounds **5a–q**. The enzymic inhibitory results revealed that most of these compounds showed significant improvement of PAK1 inhibitory activity compared to the lead (Table 1). Among **5a–e**, the methyl substitution showed no contribution to inhibitory activity, and 3-methyl (**5d**) displayed relative benefit. **5e** and **5f** with 4-site substitution presented an activity decrease, suggesting that 4-site substitution has a negative effect on affinity, which was further confirmed by **5g**, **5j** and **5n**. Next, some halogen substitution derivatives (**5g–l** and **5o**) were synthesised, but these compounds had no significant improvement in activity. Trifluoromethyl group was incorporated into 3- and 4 site respectively to obtain compounds **5m** and **5n**. Intriguingly, **5m** showed higher inhibitory potency with IC_50_ value of 2.02 μM.

According to the above SAR analysis, we hypothesised the substitution groups at 6-site of tetrahydrothieno [2,3-c]pyridine might have a huge influence on affinity. To confirm this suppose, we synthesised compounds **7a–n** keeping the 3-trifluoromethylphenyl substitution (Table 2). The results revealed that the introduction of aromatic substituents (**7a–c**) has an adverse effect on affinity. Subsequently, some simple alkyl substituents (**7d–i**) were also discussed. Among them, only compound **7d** showed a slight improvement in activity compared to **5m**. Lastly, we further designed some cycloalkane substitution derivatives (**7j–n**). Unexpectedly, the cyclopropyl substitution (**7j**) showed a significant elevation in activity with IC_50_ value of 0.21 μM. As the group size increases, the activity decreased rapidly.

**Table 2.** The PAK1 inhibitory activity of compounds **7a–n**.
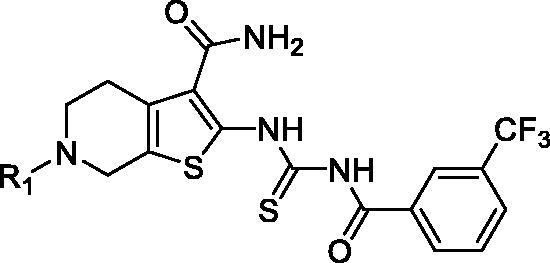

Compound	R_1_	Kinase inhibitory activity (IC_50_, μM)^a^
		PAK1
**7a**	2-methylbenzoyl	10.25 ± 1.13
**7b**	4-chlorobenzoyl	13.18 ± 1.39
**7c**	2-chlorobenzoyl	9.37 ± 1.03
**7d**	acetyl	1.15 ± 0.97
**7e**	isopropionyl	3.08 ± 0.29
**7f**	3-methylbutanoyl	3.87 ± 0.35
**7g**	3-chloropropanoyl	5.75 ± 0.63
**7h**	(E)-but-2-enoyl	4.14 ± 0.51
**7i**	methacryloyl	3.42 ± 0.33
**7j**	cyclopropanecarbonyl	0.21 ± 0.05
**7k**	cyclobutanecarbonyl	0.68 ± 0.07
**7l**	cyclopropanecarbonyl	1.09 ± 0.09
**7m**	cyclohexanecarbonyl	4.55 ± 0.51
**7n**	tetrahydro-2H-pyran-4-carbonyl	4.87 ± 0.65
**FRAX597**	–	0.023 ± 0.006

^a^Each compound was tested in triplicate; the data are presented as the mean ± SD.

### 2.4. Docking and molecular dynamic (MD) simulation

**Figure 3.**
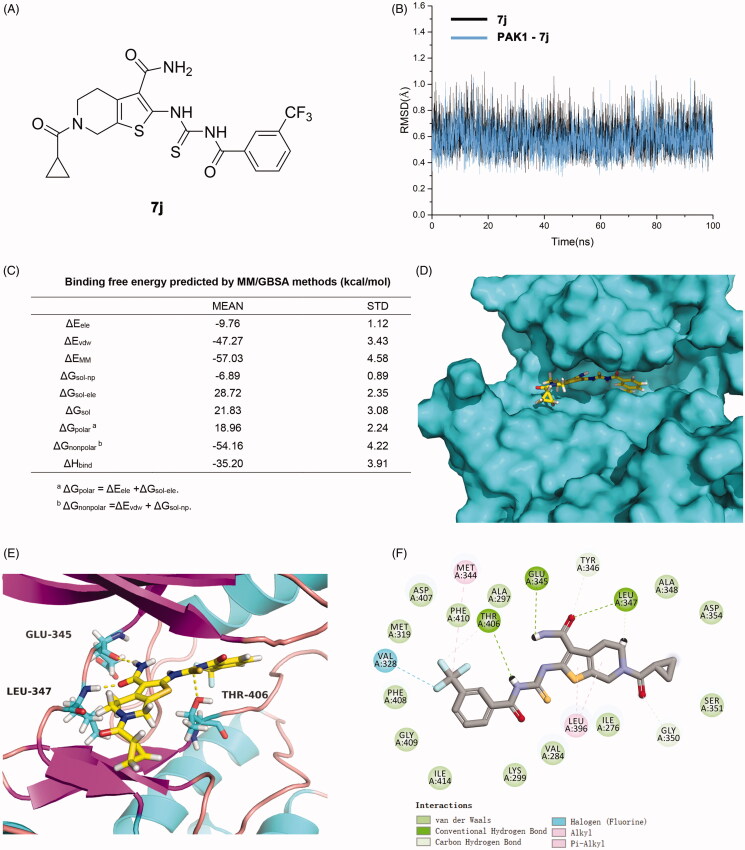
**7j** is a novel potent PAK1 inhibitor. (A) The structure of **7j**; (B) The time evolution of RMSD of backbone atoms for the residues around 5 Å of **7j** and heavy atoms of **7j**; (C) The binding free energy calculated by MM/GBSA methods (kcal/mol) of **7j** and PAK1. (D) The p interaction surface; (E, F). The detailed interactions between **7j** and PAK1.

To explore the binding pose of **7j** and PAK1, a computational study including molecular docking, molecular dynamics simulation and binding free energy calculation were performed. The root-mean-square deviation (RMSD) of the heavy ligand atoms and the backbone atom of protein around 8 Å ligand were assessed in 100 ns MD simulation, the RMSD fluctuated between 0.4 and 1.0 indicated the system was a well-behaved setup (Figure 3(B)). The binding free energy was −35.20 kcal/mol, the nonpolar term (−54.16 kcal/mol) played a primary role in **7j** binding to PAK1 (Figure 3(C)). Furthermore, a detailed view of the interactions was displayed in Figure 3(D–F). As presented, the tetrahydrothieno[2,3-c]pyridine scaffold of **7j** located the ATP-binding site and the formamide moiety at 3-site initiated two conserved hydrogen bonds with residues Glu345 and LEU347 at the kinase hinge. Additionally, a key hydrogen bond was observed between Thr406 and imide of linker, and the 3-trifluoromethylphenyl occupied the hydrophobic pocket II by hydrophobic interactions. An additional halogen bond was formed by the trifluoromethyl and residue Val328 which is a gate control residue at the activity loop. The cyclopropanecarbonyl positioned the hydrophobic entrance of kinase hinge. Collectively, **7j** is potent ATP-competing binding PAK1 inhibitor.

### 2.5 7j inhibits PAK1 activity in MDA-MB-231 cells

**Figure 4.**
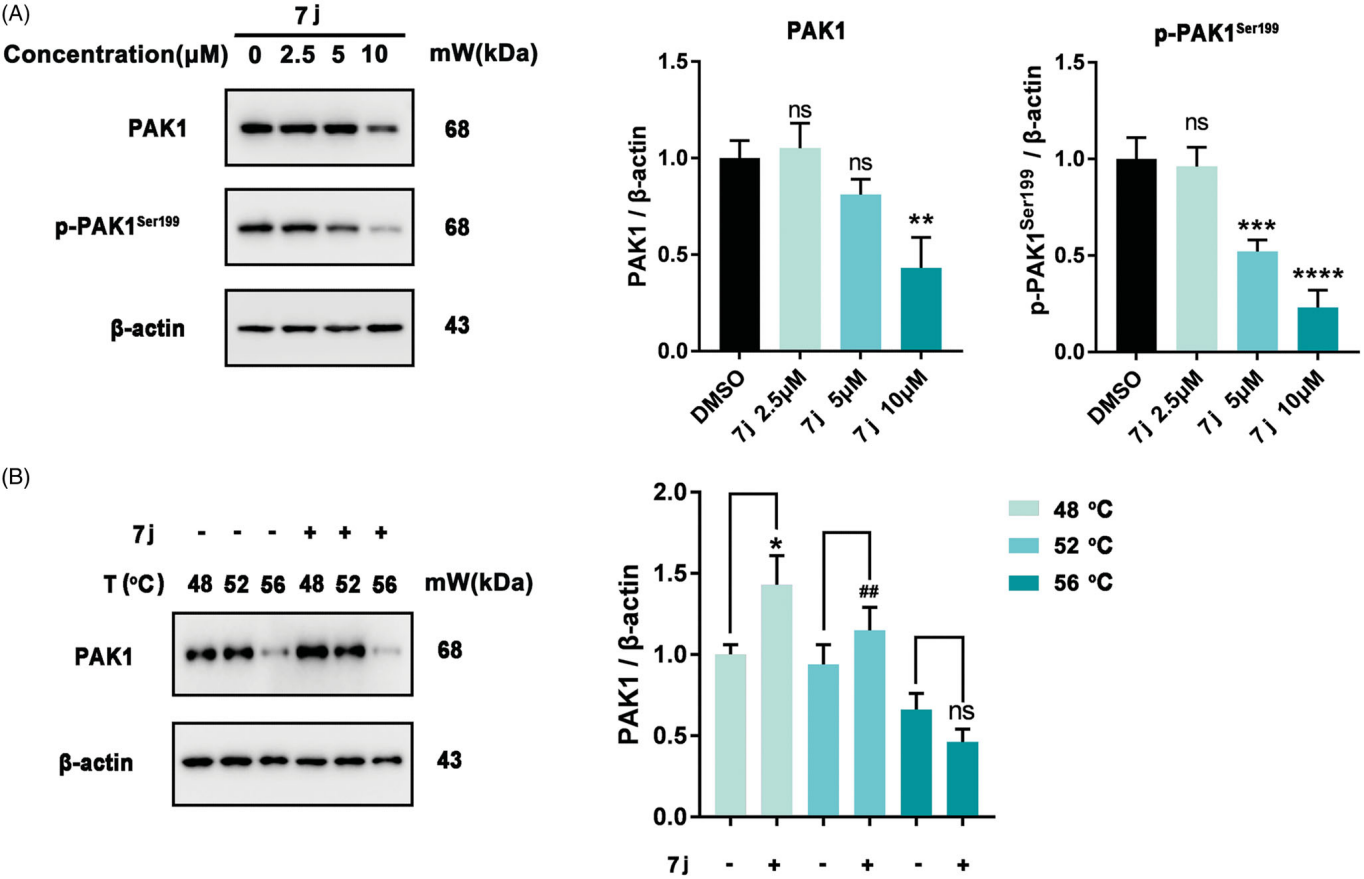
**7j** inhibited PAK1 activity in MDA-MB-231 cells. (A) Western blot analysis of PAK1, p-PAK1^Ser199^ in MDA-MB-231 cells treated with 2.5, 5, 10 µM of **7j** for 48 h. Relative PAK1 and p-PAK1^Ser199^ expression levels were quantified by normalisation to β-actin. **p < .01, ***p < .001 and ****p < .0001, compared to DMSO treated Control. (B) CETSA assay of PAK1 after **7j** treatment. Relative PAK1 expression levels were quantified by normalisation to β-actin. *p < .05, compared to DMSO treated Control at 48 °C. ^##^*p* < .01, compared to DMSO treated Control at 52 °C.

Next, to confirm whether **7j** could inhibit PAK1 activity in MDA-MB-231 cells, we firstly detected the expression and phosphorylation of PAK1 after **7j** treatment. As shown in Figure 4(A), 7j did not affect PAK1 expression in smaller dose (2.5 μM and 5 μM), but suppressed PAK1 expression in a larger dose (10 μM). At the same time, **7j** inhibited the phosphorylation of PAK1 at Ser199, which confirmed the inhibition of PAK1 activity after **7j** treatment. In addition, we performed CETSA assay to investigate whether **7j** could directly bind to PAK1. The results demonstrated that as the temperature increasing, **7j** could stabilise PAK1, which proved that **7j** could directly bind to PAK1 (Figure 4(B)). Taken together, **7j** could inhibit PAK1 activity in MDA-MB-231 cells.

### 2.6 7j inhibits MDA-MB-231 cell proliferation and induces G2/M cell cycle arrest

**Figure 5.**
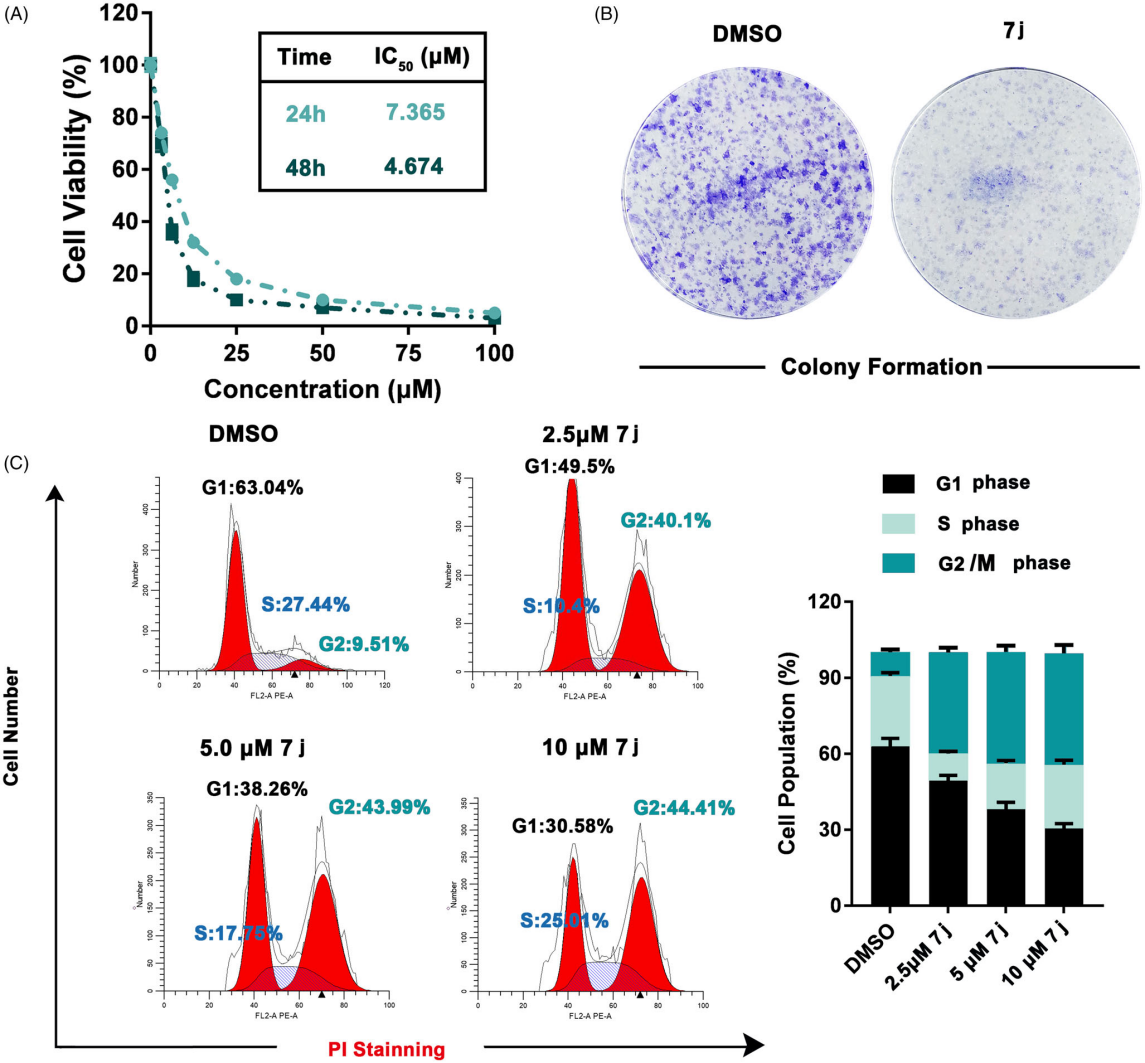
**7j** inhibited MDA-MB-231 cells proliferation and induced G2/M cell cycle arrest. (A) Cell viability were measured by MTT assay after **7j** treated for 24 h and 48 h. (B) Colony formation assay of DMSO or **7j** treated MDA-MB-231 cells. (C) MDA-MB-231 cells treated with 2.5, 5, 10 μM **7j** for 48 h and subjected to cell cycle analysis following treatment with propidium iodide.

To further explore the *in vitro* activity of **7j**, we firstly detected the effect of **7j** on MDA-MB-231 cells proliferation. As show in Figure 5(A,B), **7j** obviously inhibited the proliferation and colony formation ability of MDA-MB-231 cells. Considering the effect of PAK1 on cell cycle progression, we next evaluated the cell cycle distribution after **7j** treatment. The results demonstrated that **7j** induced obviously G2/M cell cycle arrest (Figure 5(C)). Taken together, **7j** suppressed MDA-MB-231 cell proliferation and induces G2/M cell cycle arrest.

### 2.7. 7j induced G2/M cell cycle arrest via PAK1 regulated cdc25c/cdc2 pathway

**Figure 6.**
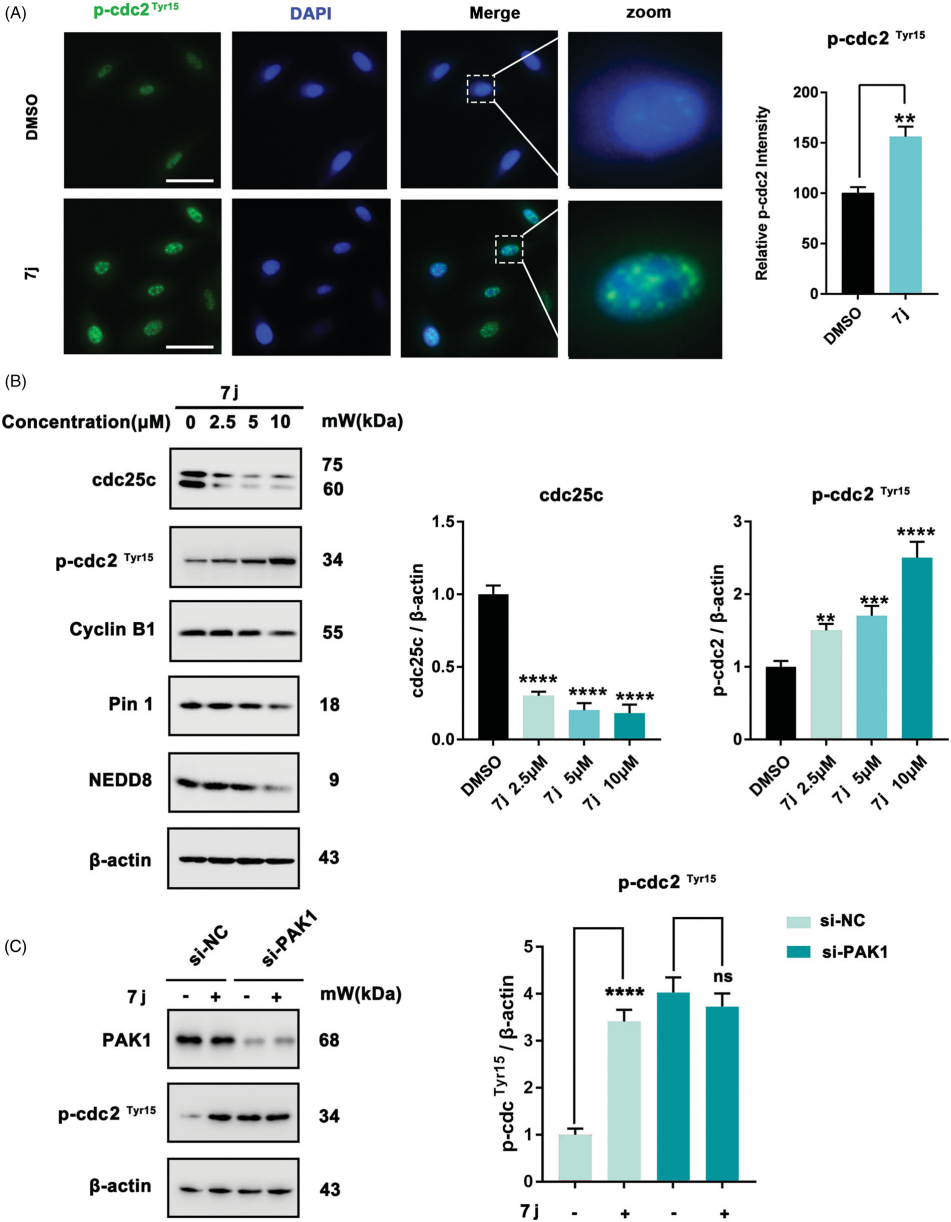
**7j** induces G2/M cell cycle arrest via cdc25c/cdc2 pathway. (A) Representative immunofluorescence images of p-cdc2 in MDA-MB-231 cells treated with DMSO (control) or 5 µM of **7j** for 48 h. The nuclei was labelled with DAPI (blue). Relative p-cdc2 intensity was quantified by Image J software, **p < .01. Scale bar = 50 μm. (B) Western blot analysis of cdc25c, p-cdc2^Tyr^^15^, CyclinB1, Pin1 and NEDD8 in MDA-MB-231 cells treated with 2.5, 5, 10 µM of **7j** for 48 h. Relative cdc25c, p-cdc2 ^Tyr^^15^ expression levels were quantified by normalisation to β-actin. **p < .01, ***p < 0.001 and ****p < .0001, compared to DMSO treated Control. (C) MDA-MB-231 cells transfected with si-NC or si-PAK1 and then treated with 5 μM **7j** for 48 h, respectively. The expression of PAK1 and p-cdc2Tyr15 were detected by western blot. Relative p-cdc2^Tyr^^15^ expression levels were quantified by normalisation to β-actin. ****p < .0001, compared to si-NC treated Control. ns, not significance, compared to si-PAK1 treated Control.

Subsequently, to detect the mechanism of **7j**-induced cell cycle arrest in MDA-MB-231 cells, we firstly measured the expression of p-cdc2^Tyr15^ which always be inhibited when cells entry into G2/M cell cycle. As shown in Figure 6(A), 7j obviously increased p-cdc2^Tyr15^ expression which demonstrated the inhibition of cdc2. Since cdc25c could active cdc2 by inducing cdc2 dephosphorylation. We next investigated the expression level of cdc25c and cyclinB which is the regulatory subunit of cdc2. And we also detected the expression of Pin1 and NEDD8 which also involved in cell cycle regulation^17^^,^^18^. The results revealed that **7j** could decrease the expression of cdc25c, cyclinB1, Pin1 and NEDD8 (Figure 6(B)). Next, the knockdown of PAK1 was performed to detect whether **7j** induced G2/M cell cycle arrest via PAK1. After PAK1 knockdown, **7j** almost did not affect the phosphorylation of p-cdc2 at Tyr15, and this confirmed that the increase of p-cdc2^Tyr15^ after **7j** treatment was mainly induced by PAK1 inhibition (Figure 6(C)). Collectively, these results demonstrated that **7j** induced G2/M cell cycle arrest via PAK1 regulated cdc25-cdc2 inhibition.

### 2.8. 7j inhibited MAPK-ERK and MAPK-JNK pathways

**Figure 7.**
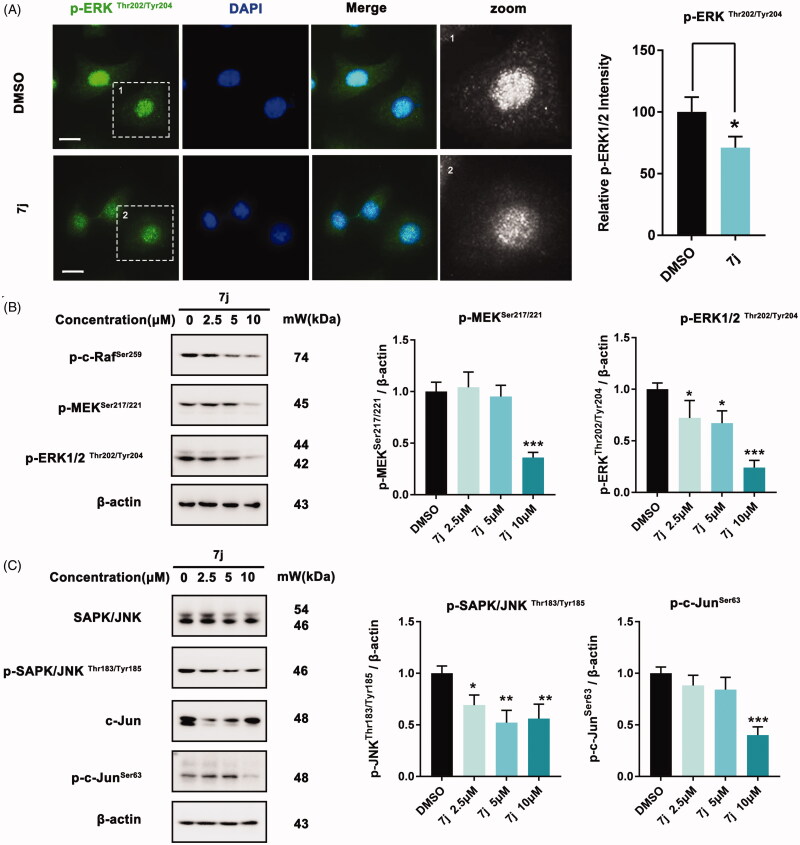
**7j** inhibits MAPK-ERK and MAPK-JNK activation. (A) Representative immunofluorescence images of p-ERK1/2 in MDA-MB-231 cells treated with DMSO (control) or 5 µM of **7j** for 48 h. The nuclei was labelled with DAPI (blue). Relative p-ERK1/2 intensity was quantified by Image J software, *p < .05. Scale bar = 20 μm. (B) Western blot analysis of p-c-Raf ^Ser259^, p-MEK1/2^Ser217/221^, and p-ERK1/2^Thr202/Tyr204^ in MDA-MB-231 cells treated with 2.5, 5, 10 µM of **7j** for 48 h. Relative p-MEK1/2^Ser217/221^, and p-ERK1/2^Thr202/Tyr204^ expression levels were quantified by normalisation to β-actin. *p < .05, and ***p < .001, compared to DMSO treated Control. (C) Western blot analysis of JNK, p-JNK, c-Jun and p-c-Jun in MDA-MB-231 cells treated with 2.5, 5, 10 µM of **7j** for 48 h. Relative p-JNK, and p-c-Jun expression levels were quantified by normalisation to β-actin. *p < .05, **p < .01 and ***p < .001, compared to DMSO treated Control.

Considering that MAPK-ERK and MAPK-JNK are two classical pathways that contribute to PAK1 regulated cell proliferation^19^, we next evaluated whether **7j** could affect these pathways. Firstly, we examined the expression and distribution of p-ERK1/2^Thr202/Tyr204^ in MDA-MB-231 after **7j** treatment. As shown in Figure 7(A), 7j inhibited its expression and nucleus distribution, which confirmed **7j** impede MAPK-ERK activation. Next, we assessed the phosphorylation of p-c-Raf, p-MEK1/2 and p-ERK1/2 and the results also demonstrated that **7j** suppressed MAPK-ERK pathway (Figure 7(B)). In addition, we investigated the expression of JNK and c-Jun as well as their phosphorylation. The results illuminated that **7j** inhibited the activation of MAPK-JNK pathway with the decrease of JNK and c-Jun phosphorylation (Figure 7(B)). In short, **7j** could inhibit MAPK-ERK and MAPK-JNK cascade.

## 3. Conclusion

**Figure 8.**
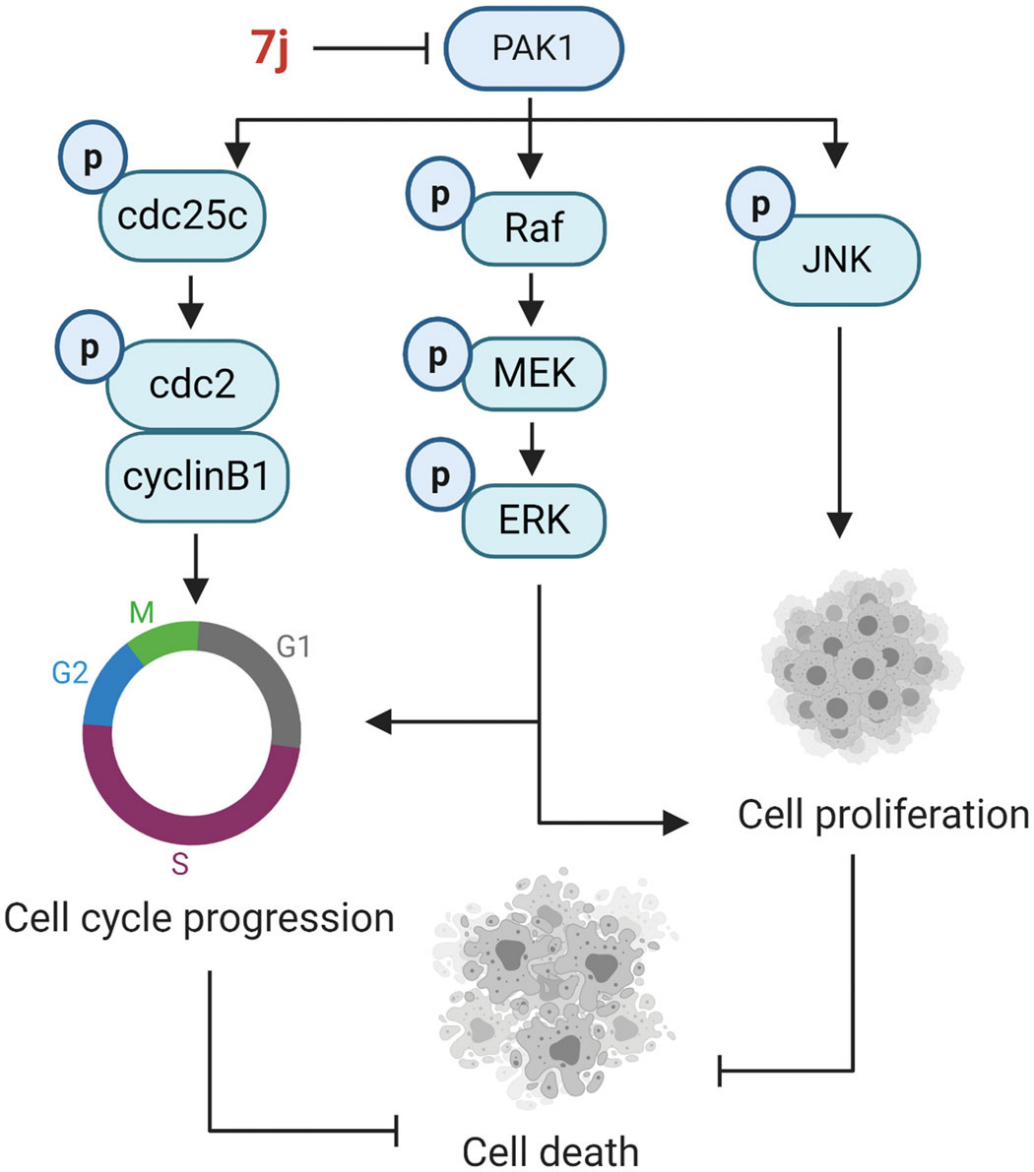
The schematic model of **7j**-induced cell death pathways, associated with cell cycle arrest and proliferation inhibition.

In summary, using a high-throughput virtual screening strategy, we found the lead (**2169–1087**) with tetrahydrothieno [2,3-c]pyridine scaffold. Based on structure-based optimisation, a series of novel tetrahydrothieno [2,3-c]pyridine substitued benzoyl thiourea derivatives was designed, synthesised and screened for inhibitory activity as novel PAK1 inhibitors. The results revealed **7j** displayed favourable effect in the PAK1 kinase assay and antiproliferative assay. Although **7j** is over 10-fold less PAK1 inhibitory activity in comparison to the standard FRAX597, the scaffold was first reported as PAK1 inhibitor, which enriched the few PAK1 inhibitor skeleton types. In addition, the molecular docking and MD simulations experiments have revealed that the binding pose of **7j** with PAK1 is typical. *In vitro*, **7j** could bind and inhibit PAK1 activity, and induced PAK1 inhibition regulated cell death. Of note, **7j** induced G2/M cell cycle arrest via PAK1-cdc25c-cdc2 pathway, and **7j** also inhibited PAK1 regulated MAPK-ERK and MAPK-JNK pathway which contribute to cell death (Figure 8). Together, these results demonstrated that **7j** is a novel potent PAK1 inhibitor, which may provide a candidate drug for future cancer therapy.

## 4. Experimental

### 4.1. Materials and measurements

All commercially available reagents and solvents were used as received. 1H-NMR spectra were recorded at 400 MHz and 13 C-NMR data were collected at 100 MHz with complete proton decoupling. ESI-HRMS spectra of all compounds were recorded by Synapt G2-SiTM (Q-TOF-MS) equipped with a high-pressure liquid chromatography (Waters Acquity I-ClassTM). Flash column chromatography was carried out on silica gel (300–400 mesh, Qingdao Marine Chemical Ltd, Qingdao, China). Thin layer chromatography (TLC) was performed on TLC silica gel 60 F254 plates. Melting points were uncorrected and determined on Shenguang melting point apparatus (SGW X-4). The purities of all final compounds were determined by HPLC to be above 95%. HPLC instrument: Waters 2695/2996 HPLC (Column: YMC-Pack ODS-A C18 (4.6 × 150 mm, 5 μm), S/N：109DA80059. Elution: MeOH in water; Flow rate: 1.0 ml/min.

### 4.2. Synthesis

#### 4.2.1. Synthesis of intermediate 3

To a solution of 4-piperidone hydrochloride (1.54 g, 10 mmol) in dichloromethane (30 ml), triethylamine (2.02 g, 20 mmol) was added at room temperature, after 30 min, Di-*tert*-butyl dicarbonate (3.27 g, 15 mmol) was added, and stirred for additional 16 h. The residue was washed with water (3 × 30 ml), the combined organic layers were washed with saturated aqueous sodium bicarbonate and brine, and then dried over anhydrous sodium sulphate. After removing the solvent under reduced pressure to give the crude product. The purity was prepared by re-crystallization from ethyl alcohol, as a white solid, yield 79%. tert-butyl 4-oxopiperidine-1-carboxylate (**3**) ^1^H-NMR (400 MHz, CDCl_3_), δ(ppm): 3.72 (4H, t, *J* = 6.1 Hz), 2.44 (4H, t, *J* = 6.1 Hz), 1.49 (9H, s); ^13 ^C-NMR (100 MHz, DMSO-*d*^6^), δ(ppm): 207.7, 154.5, 80.4, 42.9, 42.9, 41.1, 41.1, 28.4, 28.4, 28.4

#### 4.2.2. Synthesis of intermediate 4

To a solution of tert-butyl 4-oxopiperidine-1-carboxylate (30.0 mmol), 2-cyanoacetamide (30.0 mmol), and sulphur (30.0 mmol) in anhydrous ethanol (200 ml) was added morpholine (30.0 mmol). The resulting was allowed to reflux for 8 h. The suspension was cooled to ambient temperature and filtered through a pad of Celite, and washed with absolute ethyl alcohol (30 ml x 3). The combined filtrate was concentrated to dryness. The crude product was diluted by 95% ethyl alcohol (30 ml), and filtered to give the product as a white solid. ^1^H-NMR (400 MHz, DMSO-*d_6_*), δ(ppm): 6.98 (2H, s), 6.59 (2H, brs), 4.27 (2H, s), 3.50 (2H, t, *J* = 5.4 Hz), 2.67 (2H, t, *J* = 5.4 Hz), 1.42 (9H, s); ^13 ^C-NMR (100 MHz, DMSO-*d_6_*), δ(ppm): 168.0, 160.1, 154.2, 129.8, 112.6, 107.7, 79.6, 42.5, 41.9, 28.5, 28.5, 28.5, 26.5;

#### 4.2.3. General procedure synthesis of compounds 5a–q

To a solution of KSCN (117.0 mg, 1.2 mmol) in acetonitrile (10 ml), a solution of R_1_COCl (1.2 eq) in dichloromethane (2 ml) was added dropwise at room temperature. The mixture was allowed to heat reflux for 10 min. And a solution of intermediate **4** in acetonitrile (10 ml) was added, refluxed for additional 6 h. The suspension was cooled to ambient temperature and filtered through a pad of celite, and washed with absolute ethyl alcohol to afford the product as a yellow solid.

Tert-butyl-2–(3-benzoylthioureido)-3-carbamoyl-4,7-dihydrothieno[2,3-c]pyridine-6(5H)-carboxylate (**5a**)

Yellow solid, yield 83%. ^1^H-NMR (400 MHz, DMSO-*d_6_*), δ(ppm): 14.46 (1H, s), 11.73 (1H, s), 7.97 (2H, d, *J* = 6.6 Hz), 7.71 (1H, br. s), 7.66 (2H, d, *J* = 6.6 Hz), 7.53 (1H, t, *J* = 6.7 Hz), 7.35 (1H, br. s), 4.52 (2H, s), 3.57 (2H, t, *J* = 5.0 Hz), 2.78 (2H, t, *J* = 5.0 Hz), 1.44 (9H, s); ^13 ^C-NMR (100 MHz, DMSO-*d_6_*), δ(ppm): 174.6, 167.5, 166.4, 154.2, 141.5, 133.6, 132.5, 129.2, 129.2, 129.0, 128.9, 128.9, 125.2, 122.8, 79.7, 43.0, 42.3, 28.5, 28.5, 28.5, 25.5; HRMS (ESI)+ calculated for C_21_H_25_N_4_O_4_S_2_, [M + H]^+^: m/z 461.1317, found 461.1319.

Tert-butyl-3-carbamoyl-2–(3-(4-methylbenzoyl)thioureido)-4,7-dihydrothieno[2,3-c]pyridine-6(5H)-carboxylate (**5b**)

Yellow solid, yield 74%. ^1^H-NMR (400 MHz, DMSO-*d_6_*), δ(ppm): 14.47 (1H, s), 11.63 (1H, s), 7.89 (2H, d, *J* = 8.0 Hz), 7.66 (1H, br. s), 7.37 (1H, br. s), 7.34 (2H, d, *J* = 8.0 Hz), 4.51 (2H, s), 3.57 (2H, t, *J* = 5.0 Hz), 2.79 (2H, t, *J* = 5.0 Hz), 2.39 (3H, s), 1.44 (9H, s); ^13 ^C-NMR (100 MHz, DMSO-*d_6_*), δ(ppm): 174.3, 170.7, 166.3, 154.2, 141.3, 135.5, 128.9, 127.8, 127.3, 126.1, 125.2, 122.7, 79.7, 43.0, 42.3, 36.9, 28.5, 28.5, 28.5, 25.4; HRMS (ESI)+ calculated for C_22_H_27_N_4_O_4_S_2_, [M + H]^+^: m/z 475.1474, found 475.1477.

Tert-butyl-3-carbamoyl-2–(3-(2-methylbenzoyl)thioureido)-4,7-dihydrothieno[2,3-c]pyridine-6(5H)-carboxylate (**5c**)

Yellow solid, yield 87%. ^1^H-NMR (400 MHz, DMSO-*d_6_*), δ(ppm): 14.38 (1H, s), 11.84 (1H, s), 7.69 (1H, br. s), 7.50 (1H, d, *J* = 7.4 Hz), 7.44 (1H, t, *J* = 7.4 Hz), 7.33 (1H, br. s), 7.31 (1H, d, *J* = 8.4 Hz), 7.29 (1H, t, *J* = 8.4 Hz), 4.51 (2H, s), 3.57 (2H, t, *J* = 5.0 Hz), 2.79 (2H, t, *J* = 5.0 Hz), 2.39 (3H, s), 1.44 (9H, s); ^13 ^C-NMR (100 MHz, DMSO-*d_6_*), δ(ppm): 174.5, 169.5, 166.4, 154.2, 141.6, 136.5, 134.4, 131.4, 131.1, 128.9, 128.6, 125.9, 125.1, 122.6, 79.7, 43.0, 42.3, 28.5, 28.5, 28.5, 25.5, 19.9; HRMS (ESI)+ calculated for C_22_H_27_N_4_O_4_S_2_, [M + H]^+^: m/z 475.1474, found 475.1476.

Tert-butyl-3-carbamoyl-2–(3-(3-methylbenzoyl)thioureido)-4,7-dihydrothieno[2,3-c]pyridine-6(5H)-carboxylate (**5d**)

Yellow, yield 86%. ^1^H-NMR (400 MHz, DMSO-*d_6_*), δ(ppm): 14.46 (1H, s), 11.66 (1H, s), 7.83 (1H, s), 7.76 (1H, d, *J* = 7.5 Hz), 7.67 (1H, br. s), 7.47 (1H, d, *J* = 7.5 Hz), 7.42 (1H, t, *J* = 7.5 Hz), 7.37 (1H, br. s), 4.51 (2H, s), 3.57 (2H, t, *J* = 5.0 Hz), 2.79 (2H, t, *J* = 5.0 Hz), 2.39 (3H, s), 1.44 (9H, s); ^13 ^C-NMR (100 MHz, DMSO-*d_6_*), δ(ppm): 174.7, 167.5, 166.3, 154.2, 141.4, 138.3, 134.2, 132.3, 129.6, 129.0, 128.8, 126.3, 125.2, 122.7, 79.7, 43.0, 42.3, 28.5, 28.5, 28.5, 25.4, 21.3; HRMS (ESI)+ calculated for C_22_H_27_N_4_O_4_S_2_, [M + H]^+^: m/z 475.1474, found 475.1474.

Tert-butyl-3-carbamoyl-2–(3-(2,4,6-trimethylbenzoyl)thioureido)-4,7-dihydrothieno[2,3-c]pyridine-6(5H)-carboxylate(**5e**)

Yellow solid, yield 88%. ^1^H-NMR (400 MHz, DMSO-*d_6_*), δ(ppm): 14.41 (1H, s), 11.92 (1H, s), 7.68 (1H, br. s), 7.63 (1H, d, *J* = 7.5 Hz), 7.33 (1H, br. s), 6.92 (2H, s), 4.51 (2H, s), 3.57 (2H, t, *J* = 5.0 Hz), 2.79 (2H, t, *J* = 5.0 Hz), 2.26 (3H, s), 2.22 (6H, s), 1.44 (9H, s); ^13 ^C-NMR (100 MHz, DMSO-*d_6_*), δ(ppm): 174.3, 170.3, 166.4, 154.2, 141.7, 139.2, 134.4, 134.4, 133.5, 128.9, 128.4, 128.4, 125.2, 122.6, 79.7, 43.0, 42.3, 28.5, 28.5, 28.5, 25.4, 21.2, 19.4, 19.4; HRMS (ESI)+ calculated for C_24_H_31_N_4_O_4_S_2_, [M + H]^+^: m/z 503.1787, found 503.1788.

Tert-butyl-3-carbamoyl-2–(3-(4-methoxybenzoyl)thioureido)-4,7-dihydrothieno[2,3-c]pyridine-6(5H)-carboxylate (**5f**)

Yellow solid, yield 81%. ^1^H-NMR (400 MHz, DMSO-*d_6_*), δ(ppm): 14.48 (1H, s), 11.84 (1H, s), 8.00 (2H, d, *J* = 8.8 Hz), 7.66 (1H, br. s), 7.37 (1H, br. s), 7.06 (2H, d, *J* = 8.8 Hz), 4.51 (2H, s), 3.86 (3H, s), 3.57 (2H, t, *J* = 5.0 Hz), 2.79 (2H, t, *J* = 5.0 Hz), 1.44 (9H, s); ^13 ^C-NMR (100 MHz, DMSO-*d_6_*), δ(ppm): 174.8, 166.7, 166.3, 163.7, 154.2, 141.4, 131.5, 131.5, 128.9, 125.1, 124.1, 122.8, 114.2, 114.2, 79.7, 56.1, 43.0, 42.3, 28.5, 28.5, 28.5, 25.5; HRMS (ESI)+ calculated for C_22_H_27_N_4_O_5_S_2_, [M + H]^+^: m/z 491.1423, found 491.1425.

Tert-butyl-2–(3-(4-bromobenzoyl)thioureido)-3-carbamoyl-4,7-dihydrothieno[2,3-c]pyridine-6(5H)-carboxylate (**5g**)

Yellow solid, yield 71%. ^1^H-NMR (400 MHz, DMSO-*d_6_*), δ(ppm): 14.43 (1H, s), 11.84 (1H, s), 7.90 (2H, d, *J* = 8.6 Hz), 7.75 (2H, d, *J* = 8.6 Hz), 7.67 (1H, br. s), 7.35 (1H, br. s), 4.51 (2H, s), 3.57 (2H, t, *J* = 5.0 Hz), 2.79 (2H, t, *J* = 5.0 Hz), 1.44 (9H, s); ^13 ^C-NMR (100 MHz, DMSO-*d_6_*), δ(ppm): 174.5, 166.7, 166.4, 154.2, 141.5, 131.9, 131.9, 131.7, 131.3, 131.3, 129.0, 127.5, 125.2, 122.7, 79.7, 43.0, 42.3, 28.5, 28.5, 28.5, 25.5; HRMS (ESI)+ calculated for C_21_H_24_BrN_4_O_4_S_2_, [M + H]^+^: m/z 539.0422, found 539.0425.

Tert-butyl-3-carbamoyl-2–(3-(4-fluorobenzoyl)thioureido)-4,7-dihydrothieno[2,3-c]pyridine-6(5H)-carboxylate (**5h**)

Yellow solid, yield 77%. ^1^H-NMR (400 MHz, DMSO-*d_6_*), δ(ppm): 14.32 (1H, s), 12.11 (1H, s), 7.69 (1H, br. s), 7.63 (1H, d, *J* = 7.5 Hz), 7.55 (2H, d, *J* = 7.6 Hz), 7.45 (2H, d, *J* = 7.6 Hz), 7.32 (1H, br. s), 4.52 (2H, s), 3.57 (2H, t, *J* = 5.0 Hz), 2.79 (2H, t, *J* = 5.0 Hz), 1.44 (9H, s); ^13 ^C-NMR (100 MHz, DMSO-*d_6_*), δ(ppm): 174.1, 166.5, 166.7, 166.4, 154.2, 141.7, 134.8, 132.5, 130.5, 130.0, 129.8, 128.9, 127.6, 125.2, 122.6, 79.7, 43.0, 42.3, 28.5, 28.5, 28.5, 25.4; HRMS (ESI)+ calculated for C_21_H_24_FN_4_O_4_S_2_, [M + H]^+^: m/z 479.1223, found 479.1226.

Tert-butyl-3-carbamoyl-2–(3-(2-fluorobenzoyl)thioureido)-4,7-dihydrothieno[2,3-c]pyridine-6(5H)-carboxylate (**5i**)

Yellow solid, yield 81%. ^1^H-NMR (400 MHz, DMSO-*d_6_*), δ(ppm): 14.34 (1H, s), 11.86 (1H, s), 7.71 (1H, d, *J* = 7.2 Hz), 7.69 (1H, br. s), 7.65 (1H, t, *J* = 7.2 Hz), 7.37 (1H, br. s), 7.36 (1H, t, *J* = 7.0 Hz), 7.34 (1H, d, *J* = 7.0 Hz), 4.51 (2H, s), 3.57 (2H, t, *J* = 5.0 Hz), 2.79 (2H, t, *J* = 5.0 Hz), 1.44 (9H, s); ^13 ^C-NMR (100 MHz, DMSO-*d_6_*), δ(ppm): 173.9, 166.4, 164.5, 160.6, 158.9, 154.2, 141.6, 134.6, 130.9, 129.0, 125.0, 122.6, 122.5, 116.7, 116.5, 79.7, 43.0, 42.3, 36.9, 28.5, 28.5, 28.5, 25.5; HRMS (ESI)+ calculated for C_21_H_24_FN_4_O_4_S_2_, [M + H]^+^: m/z 479.1223, found 479.1227.

Tert-butyl-3-carbamoyl-2–(3-(4-chlorobenzoyl)thioureido)-4,7-dihydrothieno[2,3-c]pyridine-6(5H)-carboxylate (**5j**)

Yellow solid, yield 77%. ^1^H-NMR (400 MHz, DMSO-*d_6_*), δ(ppm): 14.43 (1H, s), 11.84 (1H, s), 7.98 (2H, d, *J* = 8.6 Hz), 7.67 (1H, br. s), 7.61 (1H, d, *J* = 8.6 Hz), 7.36 (1H, br. s), 4.51 (2H, s), 3.57 (2H, t, *J* = 5.0 Hz), 2.79 (2H, t, *J* = 5.0 Hz), 1.44 (9H, s); ^13 ^C-NMR (100 MHz, DMSO-*d_6_*), δ(ppm): 174.5, 166.5, 166.4, 154.2, 141.5, 138.4, 131.3, 131.2, 131.2, 129.0, 128.9, 128.9, 122.7, 114.2, 114.2, 79.7, 43.0, 42.3, 28.5, 28.5, 28.5, 25.4; HRMS (ESI)+ calculated for C_21_H_24_ClN_4_O_4_S_2_, [M + H]^+^: m/z 495.0927, found 495.0931.

Tert-butyl-3-carbamoyl-2–(3-(2-chlorobenzoyl)thioureido)-4,7-dihydrothieno[2,3-c]pyridine-6(5H)-carboxylate (**5k**)

Yellow, yield 78%. ^1^H-NMR (400 MHz, DMSO-*d_6_*), δ(ppm): 14.47 (1H, s), 11.63 (1H, s), 7.90 (2H, d, *J* = 8.0 Hz), 7.67 (1H, br. s), 7.37 (1H, br. s), 7.34 (2H, d, *J* = 8.0 Hz), 4.51 (2H, s), 3.57 (2H, t, *J* = 5.0 Hz), 2.79 (2H, t, *J* = 5.0 Hz), 1.44 (9H, s); ^13 ^C-NMR (100 MHz, DMSO-*d_6_*), δ(ppm): 174.7, 167.3, 166.3, 154.2, 144.1, 141.4, 129.5, 129.3, 129.0, 125.1, 122.8, 127.5, 125.2, 122.7, 79.7, 43.0, 42.3, 28.5, 28.5, 28.5, 21.6; HRMS (ESI)+ calculated for C_21_H_24_ClN_4_O_4_S_2_, [M + H]^+^: m/z 495.0927, found 495.0931.

Tert-butyl-3-carbamoyl-2–(3-(3-chlorobenzoyl)thioureido)-4,7-dihydrothieno[2,3-c]pyridine-6(5H)-carboxylate (**5l**)

Yellow solid, yield 65%. ^1^H-NMR (400 MHz, DMSO-*d_6_*), δ(ppm): 14.42 (1H, s), 11.88 (1H, s), 8.03 (1H, s), 7.90 (1H, d, *J* = 7.7 Hz), 7.72 (1H, d, *J* = 7.8 Hz), 7.69 (1H, br. s), 7.56 (1H, dd, *J* = 7.8 7.7 Hz), 7.37 (1H, br. s), 4.51 (2H, s), 3.57 (2H, t, *J* = 5.0 Hz), 2.79 (2H, t, *J* = 5.0 Hz), 1.44 (9H, s); ^13 ^C-NMR (100 MHz, DMSO-*d_6_*), δ(ppm): 174.4, 166.4, 166.2, 154.2, 141.7, 134.5, 133.5, 133.2, 130.8, 129.0, 128.9, 127.9, 125.2, 122.7, 79.7, 43.0, 42.3, 28.5, 28.5, 28.5, 25.4; HRMS (ESI)+ calculated for C_21_H_24_ClN_4_O_4_S_2_, [M + H]^+^: m/z 495.0927, found 495.0928.

Tert-butyl-3-carbamoyl-2–(3-(3-(trifluoromethyl)benzoyl)thioureido)-4,7-dihydrothieno[2,3-c]pyridine-6(5H)-carboxylate (**5m**)

Yellow solid, yield 68%. ^1^H-NMR (400 MHz, DMSO-*d_6_*), δ(ppm): 14.45 (1H, s), 12.07 (1H, s), 8.33 (1H, s), 8.24 (1H, d, *J* = 7.8 Hz), 8.02 (1H, d, *J* = 7.8 Hz), 7.78 (2H, t, *J* = 7.8 Hz), 7.68 (1H, br. s), 7.36 (1H, br. s), 4.52 (2H, s), 3.57 (2H, t, *J* = 5.0 Hz), 2.80 (2H, t, *J* = 5.0 Hz), 1.44 (9H, s); ^13 ^C-NMR (100 MHz, DMSO-*d_6_*), δ(ppm): 174.4, 166.4, 166.2, 154.2, 141.5, 133.6, 133.4, 130.1, 129.9, 129.6, 129.2, 129.0, 128.4, 126.1, 126.0, 125.6, 125.2, 79.7, 43.0, 42.3, 28.5, 28.5, 28.5, 25.4; HRMS (ESI)+ calculated for C_22_H_24_F_3_N_4_O_4_S_2_, [M + H]^+^: m/z 529.1191, found 529.1195.

Tert-butyl-3-carbamoyl-2–(3-(4-(trifluoromethyl)benzoyl)thioureido)-4,7-dihydrothieno[2,3-c]pyridine-6(5H)-carboxylate (**5n**)

Yellow solid, yield 73%. ^1^H-NMR (400 MHz, DMSO-*d_6_*), δ(ppm): 14.44 (1H, s), 12.00 (1H, s), 8.14 (2H, d, *J* = 7.6 Hz), 7.91 (2H, d, *J* = 7.6 Hz), 7.68 (1H, br. s), 7.35 (1H, br. s), 4.52 (2H, s), 3.58 (2H, t, *J* = 5.0 Hz), 2.80 (2H, t, *J* = 5.0 Hz), 1.44 (9H, s); ^13 ^C-NMR (100 MHz, DMSO-*d_6_*), δ(ppm): 174.4, 166.5, 166.4, 154.2, 141.6, 136.6, 133.3, 132.9, 132.6, 130.2, 130.2, 129.1, 125.7, 125.7, 125.6, 122.9, 122.7, 79.7, 43.0, 42.3, 28.5, 28.5, 28.5, 25.6, 25.5; HRMS (ESI)+ calculated for C_22_H_24_F_3_N_4_O_4_S_2_, [M + H]^+^: m/z 529.1191, found 529.1193.

Tert-butyl-3-carbamoyl-2–(3-(2,3-dichlorobenzoyl)thioureido)-4,7-dihydrothieno[2,3-c]pyridine-6(5H)-carboxylate (**5o**)

Yellow solid, yield 85%. ^1^H-NMR (400 MHz, DMSO-*d_6_*), δ(ppm): 14.31 (1H, s), 12.18 (1H, s), 7.79 (2H, dd, *J* = 8.0 1.2 Hz), 7.71 (1H, br. s), 7.63 (2H, dd, *J* = 8.0 1.2 Hz), 7.48 (1H, t, *J* = 7.9 Hz), 7.32 (1H, br. s), 4.52 (2H, s), 3.57 (2H, t, *J* = 5.0 Hz), 2.81 (2H, t, *J* = 5.0 Hz), 1.44 (9H, s); ^13 ^C-NMR (100 MHz, DMSO-*d_6_*), δ(ppm): 173.8, 166.5, 165.8, 154.2, 141.7, 137.1, 132.6, 132.3, 129.1, 129.0, 128.6, 128.2, 125.4, 122.5, 79.7, 43.0, 42.3, 28.5, 28.5, 28.5, 25.6; HRMS (ESI)+ calculated for C_21_H_23_Cl_2_N_4_O_4_S_2_, [M + H]^+^: m/z 529.0538, found 529.0543.

Tert-butyl-3-carbamoyl-2–(3-(cyclohexanecarbonyl)thioureido)-4,7-dihydrothieno[2,3-c]pyridine-6(5H)-carboxylate (**5p**)

Yellow solid, yield 66%. ^1^H-NMR (400 MHz, DMSO-*d_6_*), δ(ppm): 14.20 (1H, s), 11.47 (1H, s), 7.59 (1H, br. s), 7.34 (1H, br. s), 4.48 (2H, s), 3.54 (2H, t, *J* = 5.0 Hz), 2.75 (2H, t, *J* = 5.0 Hz), 1.79 (2H, d, *J* = 12.0 Hz), 1.74 (2H, d, *J* = 12.0 Hz), 1.63 (1H, m), 1.43 (9H, s), 1.37 − 1.16 (6H, m); ^13 ^C-NMR (100 MHz, DMSO-*d_6_*), δ(ppm): 177.1, 174.8, 166.2, 154.2, 141.2, 128.9, 125.1, 122.8, 79.7, 44.1, 43.0, 42.3, 29.0, 29.0, 29.0, 28.5, 28.5, 28.5, 25.6, 25.4; HRMS (ESI)+ calculated for C_21_H_31_N_4_O_4_S_2_, [M + H]^+^: m/z 467.1787, found 467.1788.

Tert-butyl-3-carbamoyl-2–(3-(2-(thiophen-2-yl)acetyl)thioureido)-4,7-dihydrothieno[2,3-c]pyridine-6(5H)-carboxylate (**5q**)

Yellow solid, yield 89%. ^1^H-NMR (400 MHz, DMSO-*d_6_*), δ(ppm): 14.44 (1H, s), 11.78 (1H, s), 8.06 (2H, t, *J* = 8.6 Hz), 7.67 (1H, br. s), 7.37 (1H, br. s), 7.35 (2H, t, *J* = 8.6 Hz), 4.51 (2H, s), 3.57 (2H, t, *J* = 5.0 Hz), 2.79 (2H, t, *J* = 5.0 Hz), 2.26 (3H, s), 2.22 (6H, s), 1.44 (9H, s); ^13 ^C-NMR (100 MHz, DMSO-*d_6_*), δ(ppm): 174.3, 170.3, 166.4, 154.2, 141.7, 139.2, 134.4, 134.4, 133.5, 128.9, 128.4, 128.4, 125.2, 122.6, 79.7, 43.0, 42.3, 28.5, 28.5, 28.5, 25.4, 21.2, 19.4, 19.4; HRMS (ESI)+ calculated for C_20_H_25_N_4_O_4_S_3_, [M + H]^+^: m/z 481.1038, found 481.1043.

#### 4.2.5. Synthesis of compound 6

To a solution of **5m** (1.0 mmol) in dichloromethane (10 ml), trifluoroacetic acid (10 ml) was added. Upon completion removing the solvent under reduced pressure, the resulting was added to water (30 ml), and basified with 1 N NaOH till pH = 9, and filtered give the product as a grey solid, yield 87%. ^1^H-NMR (400 MHz, DMSO-*d_6_*), δ(ppm): 10.15 (1H, s), 9.97 (1H, s), 8.94 (2H, s), 8.16 (1H, s), 8.15 (1H, d, *J* = 7.5 Hz), 7.92 (1H, d, *J* = 7.5 Hz), 7.71 (1H, t, *J* = 7.5 Hz), 6.65 (1H, s), 4.22 (2H, s), 3.32 (2H, t, *J* = 5.4 Hz), 3.17 (2H, t, *J* = 5.4 Hz); ^13 ^C-NMR (100 MHz, DMSO-d6), δ(ppm): 174.6, 166.4, 166.2, 141.8, 141.4, 133.6, 133.3, 130.1, 129.8, 129.8, 129.6, 129.4, 129.4, 129.2, 128.9, 127.0, 126.0, 125.6, 125.2, 123.4, 122.8, 122.5, 121.6, 43.1, 42.1, 26.4; HRMS (ESI)+ calculated for C_17_H_16_F_3_N_4_O_2_S_2_, [M + H]^+^: m/z 429.0667, found 429.0669.

#### 4.2.6. Synthesis of compound 7a–n

To a solution of **6** (214.2 mg, 0.5 mmol) and triethylamine (101.1 mg, 1 mmol) in THF (20 ml), a solution of dichloromethane (3 ml) was added dropwise at 0 °C. The mixture was stirred for 4 h at room temperature. After completion, the residue was evaporated and added 50 ml water, extracted with ethyl acetate (2 × 30 ml). The combined organic layers were washed with saturated aqueous sodium bicarbonate and brine, and then dried over anhydrous sodium sulphate. After removing the solvent under reduced pressure to give the crude product. The crude product was purified by silica sel flash chromatography (dichloromethane/methanol 10:1) as a yellow solid.

6–(2-methylbenzoyl)-2–(3-(3-(trifluoromethyl)benzoyl)thioureido)-4,5,6,7-tetrahydrothieno[2,3-c]pyridine-3-carboxamide (**7a**)

Yellow solid, yield 67%. ^1^H-NMR (400 MHz, DMSO-*d_6_*), δ(ppm): 14.50 (1H, s), 12.12 (1H, s), 8.38 (1H, s), 8.33 (1H, d, *J* = 7.8 Hz), 8.03 (1H, d, *J* = 7.8 Hz), 7.61 (2H, t, *J* = 7.8 Hz), 7.71 (1H, br. s), 7.68 (2H, d, *J* = 7.7 Hz), 7.56 (2H, d, *J* = 7.7 Hz), 7.40 (1H, br. s), 4.83 (2H, s), 3.60 (2H, t, *J* = 5.0 Hz), 2.95 (2H, t, *J* = 5.0 Hz), 2.43 (3H, s); ^13 ^C-NMR (100 MHz, DMSO-d6), δ(ppm): 174.6, 166.4, 166.2, 141.8, 141.4, 136.9, 136.4, 133.6, 133.3, 131.3, 130.1, 129.8, 129.8, 129.6, 129.4, 129.4, 129.2, 128.9, 128.4, 127.1, 127.0, 126.0, 125.6, 125.2, 123.4, 122.8, 122.5, 121.6, 43.1, 42.1, 26.4, 19.2; HRMS (ESI)+ calculated for C_25_H_21_F_3_N_4_O_3_S_2_, [M + H]^+^: m/z 547.1085, found 547.1091.

6–(4-chlorobenzoyl)-2–(3-(3-(trifluoromethyl)benzoyl)thioureido)-4,5,6,7-tetrahydrothieno[2,3-c]pyridine-3-carboxamide (**7 b**)

Yellow solid, yield 72%. ^1^H-NMR (400 MHz, DMSO-*d_6_*), δ(ppm): 14.50 (1H, s), 12.12 (1H, s), 8.38 (1H, s), 8.33 (1H, d, *J* = 7.8 Hz), 8.03 (1H, d, *J* = 7.8 Hz), 7.61 (2H, t, *J* = 7.8 Hz), 7.71 (1H, br. s), 7.68 (2H, d, *J* = 7.7 Hz), 7.56 (2H, d, *J* = 7.7 Hz), 7.40 (1H, br. s), 4.83 (2H, s), 3.60 (2H, t, *J* = 5.0 Hz), 2.95 (2H, t, *J* = 5.0 Hz); ^13 ^C-NMR (100 MHz, DMSO-*d_6_*), δ(ppm): 174.5, 171.1, 166.4, 166.2, 141.8, 141.4, 135.3, 133.6, 133.3, 130.1, 129.8, 129.8, 129.6, 129.4, 129.4, 129.2, 128.9, 127.0, 126.0, 125.6, 125.2, 123.4, 122.8, 122.5, 121.6, 43.1, 42.1, 26.4; HRMS (ESI)+ calculated for C_24_H_19_ClF_3_N_4_O_3_S_2_, [M + H]^+^: m/z 567.0539, found 567.0539.

6–(2-chlorobenzoyl)-2–(3-(3-(trifluoromethyl)benzoyl)thioureido)-4,5,6,7-tetrahydrothieno[2,3-c]pyridine-3-carboxamide (**7c**)

White solid, mp 166–169 °C, yield 76%. ^1^H-NMR (400 MHz, DMSO-*d_6_*), δ(ppm): 14.50 (1H, s), 12.12 (1H, s), 8.38 (1H, s), 8.33 (1H, d, *J* = 7.8 Hz), 8.03 (1H, d, *J* = 7.8 Hz), 7.61 (2H, t, *J* = 7.8 Hz), 7.71 (1H, br. s), 7.68 (2H, d, *J* = 7.7 Hz), 7.56 (2H, d, *J* = 7.7 Hz), 7.40 (1H, br. s), 4.83 (2H, s), 3.60 (2H, t, *J* = 5.0 Hz), 2.95 (2H, t, *J* = 5.0 Hz); ^13 ^C-NMR (100 MHz, DMSO-*d_6_*), δ(ppm): 174.5, 171.1, 166.4, 166.2, 141.8, 141.4, 135.3, 133.6, 133.3, 130.1, 129.8, 129.8, 129.6, 129.4, 129.4, 129.2, 128.9, 127.0, 126.0, 125.6, 125.2, 123.4, 122.8, 122.5, 121.6, 43.1, 42.1, 26.4; HRMS (ESI)+ calculated for C_24_H_19_ClF_3_N_4_O_3_S_2_, [M + H]^+^: m/z 567.0539, found 567.0547.

6-acetyl-2–(3-(3-(trifluoromethyl)benzoyl)thioureido)-4,5,6,7-tetrahydrothieno[2,3-c]pyridine-3-carboxamide (**7d**)

Yellow solid, mp 166–169 °C, yield 76%. ^1^H-NMR (400 MHz, DMSO-*d_6_*), δ(ppm): 14.33 (1H, s), 11.97 (1H, s), 8.26 (1H, s), 8.16 (1H, d, *J* = 7.8 Hz), 7.95 (1H, d, *J* = 7.8 Hz), 7.71 (2H, t, *J* = 7.8 Hz), 7.58 (1H, br. s), 7.26 (1H, br. s), 4.54 (2H, s), 3.62 (2H, t, *J* = 5.0 Hz), 2.81 (2H, t, *J* = 5.0 Hz), 2.04 (3H, s); ^13 ^C-NMR (100 MHz, DMSO-*d_6_*), δ(ppm): 174.5, 169.2, 166.4, 166.2, 141.8, 141.4, 133.6, 133.3, 130.1, 129.8, 129.8, 129.6, 129.4, 129.4, 129.2, 128.9, 127.0, 126.0, 125.6, 125.2, 123.4, 122.8, 122.5, 121.6, 43.1, 42.1, 26.4, 21.6; HRMS (ESI)+ calculated for C_19_H_18_F_3_N_4_O_3_S_2_, [M + H]^+^: m/z 471.0772, found 471.0774.

6-isobutyryl-2–(3-(3-(trifluoromethyl)benzoyl)thioureido)-4,5,6,7-tetrahydrothieno[2,3-c]pyridine-3-carboxamide (**7e**)

Yellow solid, yield 76%. ^1^H-NMR (400 MHz, DMSO-*d_6_*), δ(ppm): 14.45 (1H, s), 12.04 (1H, s), 8.33 (1H, s), 8.23 (1H, d, *J* = 7.8 Hz), 8.01 (1H, d, *J* = 7.8 Hz), 7.77 (2H, t, *J* = 7.8 Hz), 7.64 (1H, br. s), 7.36 (1H, br. s), 4.63 (2H, s), 3.75 (2H, t, *J* = 5.0 Hz), 2.98 (1H, m), 2.88 (2H, t, *J* = 5.0 Hz), 1.03 (6H, d, *J* = 6.2 Hz); ^13 ^C-NMR (100 MHz, DMSO-*d_6_*), δ(ppm): 175.3, 174.5, 166.4, 166.2, 141.8, 141.4, 133.6, 133.3, 130.1, 129.8, 129.8, 129.6, 129.4, 129.4, 129.2, 128.9, 127.0, 126.0, 125.6, 125.2, 123.4, 122.8, 122.5, 121.6, 43.1, 42.1, 30.8, 26.4, 19.7, 19.7; HRMS (ESI)+ calculated for C_21_H_22_F_3_N_4_O_3_S_2_, [M + H]^+^: m/z 499.1085, found 499.1087.

6–(3-methylbutanoyl)-2–(3-(3-(trifluoromethyl)benzoyl)thioureido)-4,5,6,7-tetrahydrothieno[2,3-c]pyridine-3-carboxamide (**7f**)

Yellow, yield 72%. ^1^H-NMR (400 MHz, DMSO-*d_6_*), δ(ppm): 14.41 (1H, s), 12.05 (1H, s), 8.33 (1H, s), 8.23 (1H, d, *J* = 7.8 Hz), 8.01 (1H, d, *J* = 7.8 Hz), 7.77 (2H, t, *J* = 7.8 Hz), 7.64 (1H, br. s), 7.34 (1H, br. s), 4.62 (2H, s), 3.70 (2H, t, *J* = 5.0 Hz), 2.86 (2H, t, *J* = 5.0 Hz), 2.31 (2H, d, *J* = 6.9 Hz), 2.04 (1H, m), 0.91 (6H, d, *J* = 6.4 Hz); ^13 ^C-NMR (100 MHz, DMSO-*d_6_*), δ(ppm): 174.5, 170.9, 166.4, 166.2, 141.8, 141.4, 133.6, 133.3, 130.1, 129.8, 129.8, 129.6, 129.4, 129.4, 129.2, 128.9, 127.0, 126.0, 125.6, 125.2, 123.4, 122.8, 122.5, 121.6, 43.1, 42.1, 41.7, 26.4, 23.0, 22.9, 22.6; HRMS (ESI)+ calculated for C_22_H_24_F_3_N4O_3_S_2_, [M + H]^+^: m/z 513.1242, found 513.1244.

6–(3-chloropropanoyl)-2–(3-(3-(trifluoromethyl)benzoyl)thioureido)-4,5,6,7-tetrahydrothieno[2,3-c]pyridine-3-carboxamide (**7 g**)

Yellow solid, yield 78%. ^1^H-NMR (400 MHz, DMSO-*d_6_*), δ(ppm): 14.40 (1H, s), 12.06 (1H, s), 8.33 (1H, s), 8.23 (1H, d, *J* = 7.8 Hz), 8.01 (1H, d, *J* = 7.8 Hz), 7.66 (2H, t, *J* = 7.8 Hz), 7.35 (1H, br. s), 7.26 (1H, br. s), 4.64 (2H, s), 3.83 (2H, t, *J* = 8.0 Hz), 3.72 (2H, t, *J* = 5.0 Hz), 2.96 (2H, t, *J* = 8.0 Hz), 2.87 (2H, t, *J* = 5.0 Hz); ^13 ^C-NMR (100 MHz, DMSO-*d_6_*), δ(ppm): 174.5, 172.5, 166.4, 166.2, 141.8, 141.4, 133.6, 133.3, 130.1, 129.8, 129.8, 129.6, 129.4, 129.4, 129.2, 128.9, 127.0, 126.0, 125.6, 125.2, 123.4, 122.8, 122.5, 121.6, 43.1, 42.1, 41.1, 37.1, 26.4; HRMS (ESI)+ calculated for C_20_H_19_ClF_3_N_4_O_3_S_2_, [M + H]^+^: m/z 519.0539, found 519.0540.

(E)-6-(but-2-enoyl)-2–(3-(3-(trifluoromethyl)benzoyl)thioureido)-4,5,6,7-tetrahydrothieno[2,3-c]pyridine-3-carboxamide (**7 h**)

Yellow solid, yield 65%. ^1^H-NMR (400 MHz, DMSO-*d_6_*), δ(ppm): 14.47 (1H, s), 12.11 (1H, s), 8.38 (1H, s), 8.28 (1H, d, *J* = 7.8 Hz), 8.07 (1H, d, *J* = 7.8 Hz), 7.70 (1H, br. s), 7.41 (1H, br. s), 6.73 (1H, dd, *J* = 14.4, 7.5 Hz), 6.67 (1H, d, *J* = 14.4 Hz), 4.73 (2H, s), 3.87 (2H, t, *J* = 5 Hz), 2.91 (2H, d, *J* = 5.0 Hz), 1.93 (3H, d, *J* = 7.5 Hz); ^13 ^C-NMR (100 MHz, DMSO-*d_6_*), δ(ppm): 174.5, 166.4, 166.2, 165.2, 141.8, 141.4, 133.6, 133.3, 130.1, 129.8, 129.8, 129.6, 129.4, 129.4, 129.2, 128.9, 127.0, 126.0, 125.6, 125.2, 123.4, 122.8, 122.5, 121.6, 43.1, 42.1, 26.4, 18.2; HRMS (ESI)+ calculated for C_21_H_20_F_3_N_4_O_3_S_2_, [M + H]^+^: m/z 497.0929, found 497.0933.

6-methacryloyl-2–(3-(3-(trifluoromethyl)benzoyl)thioureido)-4,5,6,7-tetrahydrothieno[2,3-c]pyridine-3-carboxamide (**7i**)

Yellow solid, yield 81%. ^1^H-NMR (400 MHz, DMSO-*d_6_*), δ(ppm): 14.51 (1H, s), 12.12 (1H, s), 8.38 (1H, s), 8.29 (1H, d, *J* = 7.8 Hz), 8.07 (1H, d, *J* = 7.8 Hz), 7.84 (2H, t, *J* = 7.8 Hz), 7.72 (1H, br. s), 7.40 (1H, br. s), 5.32 (1H, br. s), 5.15 (1H, br. s), 4.72 (2H, s), 3.77 (2H, t, *J* = 5.0 Hz), 2.92 (2H, t, *J* = 5.0 Hz), 1.97 (3H, s); ^13 ^C-NMR (100 MHz, DMSO-*d_6_*), δ(ppm): 174.5, 170.7, 166.4, 166.2, 141.8, 141.4, 133.6, 133.3, 130.1, 129.8, 129.8, 129.6, 129.4, 129.4, 129.2, 128.9, 127.0, 126.0, 125.6, 125.2, 123.4, 122.8, 122.5, 121.6, 115.7, 43.1, 42.1, 26.4, 20.6; HRMS (ESI)+ calculated for C_21_H_20_F_3_N_4_O_3_S_2_, [M + H]^+^: m/z 497.0929, found 497.0931.

6-(cyclopropanecarbonyl)-2–(3-(3-(trifluoromethyl)benzoyl)thioureido)-4,5,6,7-tetrahydrothieno[2,3-c]pyridine-3-carboxamide (**7j**)

Yellow solid, yield 61%. ^1^H-NMR (400 MHz, DMSO-*d_6_*), δ(ppm): 14.41 (1H, s), 12.03 (1H, s), 8.33 (1H, s), 8.23 (1H, d, *J* = 7.8 Hz), 8.01 (1H, d, *J* = 7.8 Hz), 7.78 (2H, t, *J* = 7.8 Hz), 7.65 (1H, br. s), 7.32 (1H, br. s), 4.64 (2H, s), 3.94 (4H, t, *J* = 3.9 Hz), 2.92 (2H, d, *J* = 5.0 Hz), 2.11 (1H, m), 0.77 (4H, br. s); ^13 ^C-NMR (100 MHz, DMSO-*d_6_*), δ(ppm): 174.5, 171.9, 166.4, 166.2, 141.8, 141.4, 133.6, 133.3, 130.1, 129.8, 129.8, 129.6, 129.4, 129.4, 129.2, 128.9, 127.0, 126.0, 125.6, 125.2, 123.4, 122.8, 122.5, 121.6, 43.1, 42.1, 26.4, 14.4, 7.47, 7.47; HRMS (ESI)+ calculated for C_21_H_20_F_3_N_4_O_3_S_2_, [M + H]^+^: m/z 497.0929, found 497.0932.

6-(cyclobutanecarbonyl)-2–(3-(3-(trifluoromethyl)benzoyl)thioureido)-4,5,6,7-tetrahydrothieno[2,3-c]pyridine-3-carboxamide (**7k**)

Yellow solid, yield 68%. ^1^H-NMR (400 MHz, DMSO-*d_6_*), δ(ppm): 14.41 (1H, s), 12.05 (1H, s), 8.33 (1H, s), 8.23 (1H, d, *J* = 7.8 Hz), 8.01 (1H, d, *J* = 7.8 Hz), 7.77 (2H, t, *J* = 7.8 Hz), 7.64 (1H, br. s), 7.34 (1H, br. s), 4.61 (2H, s), 3.70 (2H, t, *J* = 5.0 Hz), 3.43 (1H, m), 2.82 (2H, t, *J* = 5.0 Hz), 2.22 (1H, m), 2.19–2.12 (3H, m), 1.92 (1H, m), 1.76 (1H, m); ^13 ^C-NMR (100 MHz, DMSO-*d_6_*), δ(ppm): 174.5, 172.9, 166.4, 166.2, 141.8, 141.4, 133.6, 133.3, 130.1, 129.8, 129.8, 129.6, 129.4, 129.4, 129.2, 128.9, 127.0, 126.0, 125.6, 125.2, 123.4, 122.8, 122.5, 121.6, 43.5, 42.3, 37.1, 36.8, 36.8, 26.4, 17.9; HRMS (ESI)+ calculated for C_22_H_22_F_3_N_4_O_3_S_2_, [M + H]^+^: m/z 511.1085, found 511.1086.

6-(cyclopentanecarbonyl)-2–(3-(3-(trifluoromethyl)benzoyl)thioureido)-4,5,6,7-tetrahydrothieno[2,3-c]pyridine-3-carboxamide (**7 l**)

Yellow solid, yield 78%. ^1^H-NMR (400 MHz, DMSO-*d_6_*), δ(ppm): 14.44 (1H, s), 12.05 (1H, s), 8.33 (1H, s), 8.23 (1H, d, *J* = 7.8 Hz), 8.01 (1H, d, *J* = 7.8 Hz), 7.77 (2H, t, *J* = 7.8 Hz), 7.64 (1H, br. s), 7.34 (1H, br. s), 4.61 (2H, s), 3.74 (2H, t, *J* = 5.0 Hz), 2.88 (2H, t, *J* = 5.0 Hz), 2.68 (1H, m), 1.71–1.64 (4H, m), 1.37–1.33 (4H, m); ^13 ^C-NMR (100 MHz, DMSO-*d_6_*), δ(ppm): 174.5, 170.0, 166.4, 166.2, 141.8, 141.4, 133.6, 133.3, 130.1, 129.8, 129.8, 129.6, 129.4, 129.4, 129.2, 128.9, 127.0, 126.0, 125.6, 125.2, 123.4, 122.8, 122.5, 121.6, 43.5, 42.3, 40.8, 29.7, 29.7, 26.4, 26.0, 21.8; HRMS (ESI)+ calculated for C_23_H_23_F_3_N4O_3_S_2_, [M + H]^+^: m/z 525.1242, found 539.1268.

6-(cyclohexanecarbonyl)-2–(3-(3-(trifluoromethyl)benzoyl)thioureido)-4,5,6,7-tetrahydrothieno[2,3-c]pyridine-3-carboxamide (**7 m**)

Yellow solid, yield 73%. ^1^H-NMR (400 MHz, DMSO-*d_6_*), δ(ppm): 14.44 (1H, s), 12.05 (1H, s), 8.33 (1H, s), 8.23 (1H, d, *J* = 7.8 Hz), 8.01 (1H, d, *J* = 7.8 Hz), 7.77 (2H, t, *J* = 7.8 Hz), 7.64 (1H, br. s), 7.34 (1H, br. s), 4.61 (2H, s), 3.74 (2H, t, *J* = 5.0 Hz), 2.88 (2H, t, *J* = 5.0 Hz), 2.68 (1H, m), 1.71–1.64 (5H, m), 1.37–1.33 (5H, m); ^13 ^C-NMR (100 MHz, DMSO-*d_6_*), δ(ppm): 174.5, 170.0, 166.4, 166.2, 141.8, 141.4, 133.6, 133.3, 130.1, 129.8, 129.8, 129.6, 129.4, 129.4, 129.2, 128.9, 127.0, 126.0, 125.6, 125.2, 123.4, 122.8, 122.5, 121.6, 43.5, 42.3, 40.8, 29.7, 29.7, 26.4, 26.0, 26.0, 21.8; HRMS (ESI)+ calculated for C_24_H_26_F_3_N_4_O_3_S_2_, [M + H]^+^: m/z 539.1398, found 539.1398.

6-(tetrahydro-2H-pyran-4-carbonyl)-2–(3-(3-(trifluoromethyl)benzoyl)thioureido)-4,5,6,7-tetrahydrothieno[2,3-c]pyridine-3-carboxamide (**7n**)

Yellow solid, yield 71%. ^1^H-NMR (400 MHz, DMSO-*d_6_*), δ(ppm): 14.46 (1H, s), 12.03 (1H, s), 8.32 (1H, s), 8.23 (1H, d, *J* = 7.8 Hz), 8.01 (1H, d, *J* = 7.8 Hz), 7.77 (2H, t, *J* = 7.8 Hz), 7.63 (1H, br. s), 7.32 (1H, br. s), 4.39 (2H, s), 3.61 (4H, t, *J* = 3.9 Hz), 3.43 (2H, d, *J* = 5.0 Hz), 3.17 (4H, t, *J* = 3.9 Hz), 2.86 (2H, t, *J* = 5.0 Hz), 2.50 (1H, m); ^13 ^C-NMR (100 MHz, DMSO-*d_6_*), δ(ppm): 174.5, 166.4, 166.2, 163.2, 141.8, 141.4, 133.6, 133.3, 130.1, 129.8, 129.8, 129.6, 129.4, 129.4, 129.2, 128.9, 127.0, 126.0, 125.6, 125.2, 123.4, 122.8, 122.5, 121.6, 66.4, 66.4, 45.4, 44.3, 30.8, 30.8, 25.4; HRMS (ESI)+ calculated for C_23_H_24_F_3_N_4_O_4_S_2_, [M + H]^+^: m/z 541.1191, found 541.1193.

### 4.3. Molecular docking

The discovery Studio 3.5 docking program was adopted here^20^. The preparation of protein structure (PDB code 4ZY5)^21^, including adding hydrogen atoms, removing water molecules, and assigning Charmm forcefield. Goldscore was selected as the score function, and the other parameters were set as default. For each docking study, a total of 10 docking poses were retained. The root-mean square deviation (RMSD) between docking poses were calculated.

### 4.4. Molecular dynamics (MD) simulations

The MD simulation was performed by Amber 10 package^22^. The first restraining energy minimisation was carried out by the steepest descent method with 0.1 kcal/mol•Å2 restraints for all atoms of the complexes for 5000 steps. And then, we removed the restraints of ligand (only restraining the protein) to perform the second energy minimisation, and another energy minimisation was made under releasing all the restraints. 5000 steps were set for each energy minimisation. To handle the long-range Coulombic interactions, the particle mesh Ewald (PME) summation was used. The SHAKE algorithm was employed on all atoms covalently bonded to a hydrogen atom, allowing for an integration time step of 2 fs in the equilibration and subsequent production runs. The annealed programme was from 0 to 310 K for 50 ps. Under releasing all the restraints, the system was again equilibrated for 500 ps. The production phase of the simulations was run without any restraints for a total of 100 ns.

### 4.5. Cell culture, antibodies and reagents

MDA-MB-231 were purchased from American Type Culture Collection (ATCC, Manassas, VA, USA). Cells were cultured in DMEM with 10% foetal bovine serum and incubated with 5% CO2. Antibodies used in this study were as follows: cdc25c (4688, CST), pcdc2^Tyr^^15^ (4539, CST), cyclinB1 (4135, CST), PAK1 (ab223849, CST), p-PAK1^Ser199^ (ab192814, abcam), JNK (9252, CST), p-JNK (9255, CST), c-Jun (9165, CST), p-c-Jun (3270, CST), p-c-Raf^Ser259^(9421, CST), p-MEK ^Ser217/221^ (9154, CST), p-ERK ^Thr202/Tyr204^ (9101, CST), β-actin (3700, CST). MTT (M2128) was purchased from Sigma- Aldrich (St. Louis, MO, USA).

### 4.6. Cell viability assay

Cells were dispensed in 96-well plates at a density of 5 × 10^4^ cells/ml. After 24 h or 48 h incubation, cells were treated with different concentrations of compounds for the indicated time periods. Cell viability was measured by the MTT assay.

### 4.7. Immunofluorescence analysis

For immunofluorescence staining, non-specific antibody binding was blocked by incubating with PBS containing 1.5% goat serum. The MDA-MB-231 cells were sequentially incubated, starting with p-cdc2^Tyr^^15^ antibody (1:200), p-ERK1/2 ^Thr202/Tyr204^ antibody (1:200) diluted in PBS containing 1% BSA incubated overnight at 4 °C, followed by addition of fluorescent-labelled secondary antibodies for 1 h at room temperature.

### 4.8. Western blot

Cells were treated with 7j for indicated times. Both adherent and floating cells were collected. Western blot analysis was carried out. Briefly, the cell pellets were resuspended with lysis buffer consisting of Hepes 50 mM pH 7.4, Triton-X-100 1%, sodium orthovanada 2 mM, sodium fluoride 100 mM, edetic acid 1 mM, PMSF 1 mM, aprotinin (Sigma, MO, USA) 10 mg/L and leupeptin (Sigma) 10 mg/L and lysed at 4 °C for 1 h. After 12,000 rpm centrifugation for 15 min, the protein content of supernatant was determined by the Bio- Rad DC protein assay (Bio-Rad Laboratories, Hercules, CA, USA). Equal amounts of the total protein were separated by 10–15% SDS-PAGE and transferred to PVDF membranes, the membranes were soaked in blocking buffer (5% skimmed milk or BSA). Proteins were detected using primary antibodies, followed by HRP-conjugated secondary antibody and visualised by using ECL as the HRP substrate. Quantification of immunoblot was performed by Quantity One 4.4.

### 4.9. Cell cycle assay

For cell cycle detection, MDA-MB-231 cells were treated with 2.5, 5, 10 μM 7j for 48 h and then ethyl alcohol-fixed at 4 °C for 24 h. Then the cell cycle distribution were determined by flow cytometry analysis of PI staining.

### 4.10. Transfection assays

Cells were transfected with PAK1 (6361, CST) and negative control (6568, CST) siRNAs at 100 nM final concentration using Lipofectamine RNAiMAX/Lip3000 reagent (Invitrogen) according to the manufacturer’s instructions. The transfected cells were used for subsequent experiments 48 h later.

### 4.11. *The* enzymatic assay

These assays were carried out as described previously^19^. All of the enzymatic reactions were conducted at 30 °C for 40 min. The 50 µl reaction mixture contains 40 mM Tris, pH 7.4, 10 mM MgCl2, 0.1 mg/ml BSA, 1 mM DTT, 50 µM ATP, 0.2 μg/ml PAK1 and 100 uM lipid substrate. The compounds were diluted in 10% DMSO and 5 µl of the dilution was added to a 50 µl reaction so that the final concentration of DMSO is 1% in all of the reactions. The assay was performed using the Kinase-Glo Plus luminescence kinase assay kit and ADP-Glo Plus luminescence kinase assay kit. It measures kinase activity by quantitating the amount of ATP remaining in solution following a kinase reaction. The luminescent signal from the assay is correlated with the amount of ATP present and is inversely correlated with the amount of kinase activity. The IC_50_ values were calculated using nonlinear regression with normalised dose − response fit using Prism GraphPad software.

### 4.12. Cellular thermal shift assay (CETSA)

The ability of **7j** to interact with and stabilise PAK1 protein in intact cells, was analysed as described before^23^. Briefly, cells cultured in 100 × 20 mm tissue culture dishes at 90% confluence were treated with media containing DMSO or **7j** (10 μM) for 24 h. After that cells were detached with trypsin and re-suspended in PBS. The cell suspension was heated for 3 min to 48, 52, and 56 °C. Subsequently, cells were lysed using liquid nitrogen and two repeated cycles of freeze-thaw. Precipitated proteins were separated from the soluble fraction by centrifugation at 17,000 g for 20 min. Soluble proteins, collected in the supernatant, were kept at −80 °C until Western blot analysis.

### 4.13. Statistical analysis

All the presented data and results were confirmed by at least three independent experiments. The data are expressed as means ± SEM and analysed with GraphPad Prism 7.0 software. Statistical comparisons were made by One-way ANOVA and Student’s *t*-test. *p* < .05 was considered statistically significant.
